# Single‐cell RNA sequencing reveals the characteristics of cerebrospinal fluid tumour environment in breast cancer and lung cancer leptomeningeal metastases

**DOI:** 10.1002/ctm2.885

**Published:** 2022-06-09

**Authors:** Haoyu Ruan, Zhe Wang, Ziwei Sun, Jia Wei, Lei Zhang, Huanyu Ju, Ting Wang, Chao Zhang, Ming Guan, Shiyang Pan

**Affiliations:** ^1^ Department of Laboratory Medicine The First Affiliated Hospital of Nanjing Medical University Nanjing China; ^2^ Department of Physiology, Second Military Medical University Shanghai China; ^3^ Department of Laboratory Medicine Huashan Hospital Fudan University Shanghai China; ^4^ Department of Plastic and Reconstructive Surgery Shanghai Institute of Precision Medicine Shanghai Ninth People's Hospital Shanghai Jiao Tong University School of Medicine Shanghai China; ^5^ Branch of National Clinical Research Center for Laboratory Medicine Nanjing China

**Keywords:** Cerebrospinal fluid, circulating tumour cells, gene regulatory networks, leptomeningeal metastases, tumour microenvironment

## Abstract

Leptomeningeal metastases (LM) occur in patients with breast cancer (BC) and lung cancer (LC) showing exceptionally poor prognosis. The cerebrospinal fluid (CSF) tumour microenvironment (TME) of LM patients is not well defined at a single‐cell level. Based on the 10× genomics single‐cell RNA sequencing (scRNA‐seq) data from GEO database including five patient‐derived CSF samples of BC‐LM and LC‐LM, and four patient‐derived CSF samples of idiopathic intracranial hypertension (IIH) as controls, we analysed single‐cell transcriptome characteristics of CSF TME in LM patients compared to controls simultaneously and comprehensively. In addition, we performed 10× genomics scRNA‐seq on CSF cells derived from a BC‐LM patient to help generate a solid conclusion. The CSF macrophages in LM patients showing M2‐subtype signature and the emergence of regulatory T cells in LM confirmed the direction of tumour immunity toward immunosuppression. Then, the characteristics of CSF circulating tumour cells (CTCs) of breast cancer LM (BC‐LM) patients were classified into five molecular subtypes by PAM50 model. The communication between macrophages and five subtype‐specific CSF‐CTCs showed largest number of ligand‐receptor interactions. The five subtypes‐specific CSF‐CTCs showed great heterogeneities which were manifested in cell proliferation and cancer‐testis antigens expression. Gene regulatory networks (GRNs) analysis revealed that transcription factor SREBF2 was universally activated in the five subtypes‐specific CSF‐CTCs. Our results will provide inspiration on new directions of the mechanism research, diagnosis and therapy of LM.

## Graphical abstract

- Integrated analysis of characteristics of CSF tumour environment of LM patients.
- Five subtype‐specific CSF‐CTCs of breast cancer showed great heterogeneities on cell proliferation and cancer‐testis antigens expression.
- Gene regulatory networks (GRNs) highlighted transcription factor SREBF2 activated in the breast cancer CSF‐CTCs

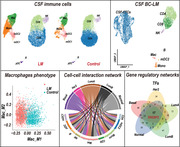

## 1 INTRODUCTION

Lung, breast and melanoma are the three most common primary tumours related with brain metastases. Breast cancer (BC) is the most common women's malignant tumours occurring brain metastasis.^1^ With the prolonged survival time of advanced BC patients, the incidence of BM is increasing and has become a major problem affecting survival. The incidence of BM is higher in HER2‐positive and triple‐negative patients, and most triple‐negative patients occur BM in the early‐stage cancer.^1^

BC‐BM included parenchymal brain metastases (PBM) and leptomeningeal metastases (LM), which can occur either separately or simultaneously. PBM refer to multiple intracranial space‐occupying lesions shown on CT or magnetic resonance imaging (MRI). LM refers to dissemination of cancer cells to the pia mater, arachnoid and subarachnoid space. A positive cerebrospinal fluid (CSF) cytology result is the gold standard for LM diagnosis. BC‐LM can present as a late‐stage complication of systemic progression or the first sign of metastatic disease.^2^ The prognosis of BC‐LM remains poor despite of advances in multimodal therapies, including surgery, radiotherapy, chemotherapy, immunotherapy and targeted therapies.^1^ There are still challenges in diagnosis and treatment of BC‐LM.

Interests always abound in understanding the mechanisms that drive LM, which are thought to originate from the seeding of circulating tumour cells (CTCs) into leptomeninges. Single‐cell RNA sequencing (scRNA‐seq) technology make us available to obtain the single‐cell transcriptome data of CSF cells. For instance, scRNA‐seq methods have fuelled the investigations of CSF‐CTCs of LC^3^ and central nervous system lymphoma,^4^ and emphasised on the CSF microenvironment of HIV infection,^5^ alzheimer's disease,^6^ multiple sclerosis^7^ and LM.^8^

In this study, we intended to analyse the characteristics of CSF tumour microenvironment (TME) of LM patients. We enrolled scRNA‐seq data of nine CSF samples from Gene Expression Omnibus (GEO) database, including five CSF samples from three breast cancer LM patients (BC‐LM) and two lung cancer LM (LC‐LM) patients.^8^ We performed 10× genomics scRNA‐seq on CSF cells derived from one BC‐LM patient. ScRNA‐seq data of four CSF samples from patients with idiopathic intracranial hypertension (IIH) served as controls.^7^ First, we concentrated on the analysis of CSF immune cells. The LM mainly affected the CSF macrophages and regulatory T cells (Tregs). Second, we focused on the characteristics of BC‐LM CSF‐CTCs. BC CSF‐CTCs were classified into five subtypes by the PAM50 model.^9^, ^10^ The analysis of ligand–receptor interaction was performed by python CellPhoneDB package^11^ to reveal the crosstalk between five subtype‐specific CSF‐CTCs and immune cells in the CSF microenvironment. Third, we intended to analyse to the heterogeneity of BC‐LM CSF‐CTCs of the five different subtypes from the aspects of cancer‐testis antigens (CTAs) expression and cell‐cycle state. Fourth, we proposed a gene regulatory networks (GRNs) analysis, and dissected the critical transcription factors (TFs) enriched for five subtype‐specific CSF‐CTCs. Thus, the comprehensive analysis of scRNA‐seq data of CSF cells derived from LM patients could help us dissect critical genes for LM tumorigenesis.

## 2 Methods

### 2.1 Data collection

The 10× genomics scRNA‐seq data of five LM patient‐derived CSF samples were from GEO (https://www.ncbi.nlm.nih.gov/geo/) database (GSE150681), including three BC LM patients (BC‐LM‐B, GSM4555888; BC‐LM‐C, GSM4555889; and BC‐LM‐E, GSM4555891) and two LC LM patients (LC‐LM‐A, GSM4555887; and LC‐LM‐D, GSM4555890).^8^ The 10× genomics scRNA‐seq data of four CSF samples from patients with IIH as controls were obtained from GSE138266 (Control 1–4, GSM4104129, GSM4104131, GSM4104132, GSM4104133).^7^ The gene expression profiling of three subtypes (one basal cluster and two luminal clusters) normal breast epithelial cells were acquired from GEO database, including 10× genomics scRNA data from GSE113196 (Normal sample 4–6, N4, N5, N6) and Fluidigm C1 scRNA data from GSE113099, GSE113127, and GSE113198 (Normal sample 1–3, N1‐N3).^12^

The accession number for original data of 10× genomics scRNA‐seq of CSF cells derived from a 63‐year‐old woman affected by breast cancer LM (BC‐LM‐F) at Huashan Hospital, Fudan University in this study is GEO: GSE202501. The consent forms and the proposed studies were approved by Institutional Review Board of Huashan Hospital (HIRB, KY2019‐002).

The single cell date of BC blood‐CTCs were obtained, including 35 blood‐CTCs sequenced by smart‐seq2 method^13^ (GSE109761), 75 blood‐CTCs sequenced by 10× genomics scRNA‐seq method^14^ (GSE139495) and 693 blood‐CTCs presented by Hydro‐Seq method.^15^

### 2.2 Filtering and normalization of scRNA‐seq data

Cells had less than 1000 counts and 300 genes were removed for following analyses (Seurat package version 4.0.4). After filtration, gene expression matrix were merged and the batch effect was removed with the R package harmony v1.0 (lambda = 1, max.iter.harmony = 20). The merged expression matrix was prepared for clustering using the Seurat 4.0.4, following the common pipeline. Uniform Manifold Approximation and Projection (UMAP) dimensionality reduction was used to project cells in two dimensions. An R project including counts and celltypes of five LM patient‐derived CSF samples and four control CSF samples are provided on figshare website (https://figshare.com, https://doi.org/10.6084/m9.figshare.19130036).

The feature plots based on R package Seurat were used to demonstrate the expression marker genes. Marker genes: T cells (T), CD3E and TRAC; CD4+ T cells (CD4): CD4 and IL7R; CD8+ T cells (CD8): CD8A, CD8B; regulatory T cells (Treg): FOXP3 and CTLA4; NK cells (NK): GNLY, PRF1, and XCL1; myeloid lineage cells: LYZ; monocyte cells (Mono): FCGR3A, S100A8; Macrophage cells (Mac): CD14, PLTP, MRC1, NLRP3 and IL1B; myeliod DC type 1 cells (mDC1): XCR1; myeliod DC type 2 cells (mDC2): FCER1A, CD1C; plasmacytoid DC cells (pDC): TNFRSF21; B cells (B): CD79A; tumour cells: EPCAM, CDH1, KRT18 and KRT19.

### 2.3 Identification of differentially expressed genes

We applied the FindAllMarkers function in R package Seurat (4.0.4) to identify differentially expressed genes (DEGs) for each cell clusters. The Wilcoxon Rank‐Sum test was applied to determine the significance of the difference. The DEGs in the cell state over the trajectory were selected for subsequent analysis by average Log_2_FoldChange ≥ 0.25, adjusted *P*‐value < 0.01.

The DEGs between CSF‐CTCs and blood‐CTCs were filtered based on the statistical threshold (Log_2_FoldChange ≥ 1, adjusted *P*‐value < 0.05), so that their expression is significantly higher in CSF‐CTCs.

FindAllMarkers function was also performed to select signature genes in different cell clusters according to the statistical threshold (log_2_FoldChange ≥  1, adjusted *P*‐value <  0.01). Signature genes need to be expressed in > 25% of cells in a cell cluster and < 5% in other cell clusters.

### 2.4 Modular score calculation

AddModuleScore function in R package Seurat (4.0.4) was applied to the calculation of the gene expression modular scores per cell. The conventional classic activated macrophages (M1‐subtype) modular included genes IL1B, IL1A, TNF, IL6, CXCL9, CXCL10, IL12A, IL12B, IL23A, FCGR1A, FCGR1B, FCGR1C, CCR7, IL8, CCL5, HLA‐DRA, IRF5, IRF1. The alternative activated macrophage (M2‐subtype) modular contained genes IL10, CD163, MARCO, MRC1, MSR1, ARG1, STAB1, TGM2, MMP7, MMP9, MMP19, TGFB1, TGFB2, TGFB3, VEGFA, FN1, CCL4, CCL22, CCL17, CCL18, IL4R, IL7R, IRF4.

### 2.5 Trajectory analysis

Pseudotime analysis was performed using R package Monocle2 (v2.18.0) to determine the dramatic translational relationships among different cells. FindAllMarkers function in R package Seurat (4.0.4) was taken to identify specific genes in each state with average log_2_Foldchange ≥ 0.25, adjusted *P*‐value < 0.01, Wilcoxon Rank‐Sum test.

### 2.6 Cell–cell communication

The python CellPhoneDB (v.2.0, https://www.cellphonedb.org/) package was applied to investigate the cell–cell communication, ligand–receptor interaction with *P*‐value < 0.05 were considered as significant.^11^

### 2.7 Identification of five subtype‐specific CSF‐CTCs of BC‐LM patients

The PAM50 model was performed to divide CSF‐CTCs of BC‐LM patients into five molecular subtypes, named as normal‐like (normal), basal‐like (basal), human epidermal growth factor receptor‐2 positive (Her2), Luminal A (LumA) and Luminal B (LumB), according to the expression of 50 signature genes^10^ by using the R package genefu (2.24.2), the SubPred_pam50 function.^9^ The 852 CSF‐CTCs were retained with score in one molecular subtype > 0.5, and the highest score minus the second highest score > 0.2.

### 2.8 Gene set variation analysis

The gene set variation analysis (GSVA) made use of the R package GSVA (1.40.1)^16^ to test the activation of the 50 hallmark gene sets the Molecular Signatures Database (https://www.gsea‐msigdb.org/gsea/msigdb).

### 2.9 Construction of GRNs and identification of hub genes

The R package SCENIC (v1.1.0) was applied to analysing the enrichment of TF‐binding motifs per cell.^17^ The co‐expressed TF–target gene pairs, enriching a TF‐binding motif and showing normalised enrichment score (NES) > 3, were considered as a significant regulon for GRN construction. The activity of each regulon in each cell was calculated by AUCell in R‐package SCENIC, which makes it possible to identify which cells have significantly higher subnetwork activity.

The hub gene in the 5 GRNs was identified by CytoHubba in Cytoscape (3.8.2).^18^ The importance of nodes was measured by using the node centrality metrics, including degree, bottleNeck, closeness, radiality, and betweenness. The top 1% nodes sorted by the centrality metrics were considered to be hub genes.

### 2.10 Quantification and statistical analysis

Differential analysis between two groups was performed using two‐sided Student's *t*‐test, or Wilcoxon Rank‐Sum test. Difference among multiple pairwise comparisons was analysed by one‐way ANOVA test or R Package Dunn.test (v1.3.5) using Kruskal–Wallis test. *P*‐value < 0.05 was considered to be significant.

## 3 RESULTS

### 3.1 CSF immune cells of LM patients exhibit a specific composition and transcriptome

**FIGURE 1:**
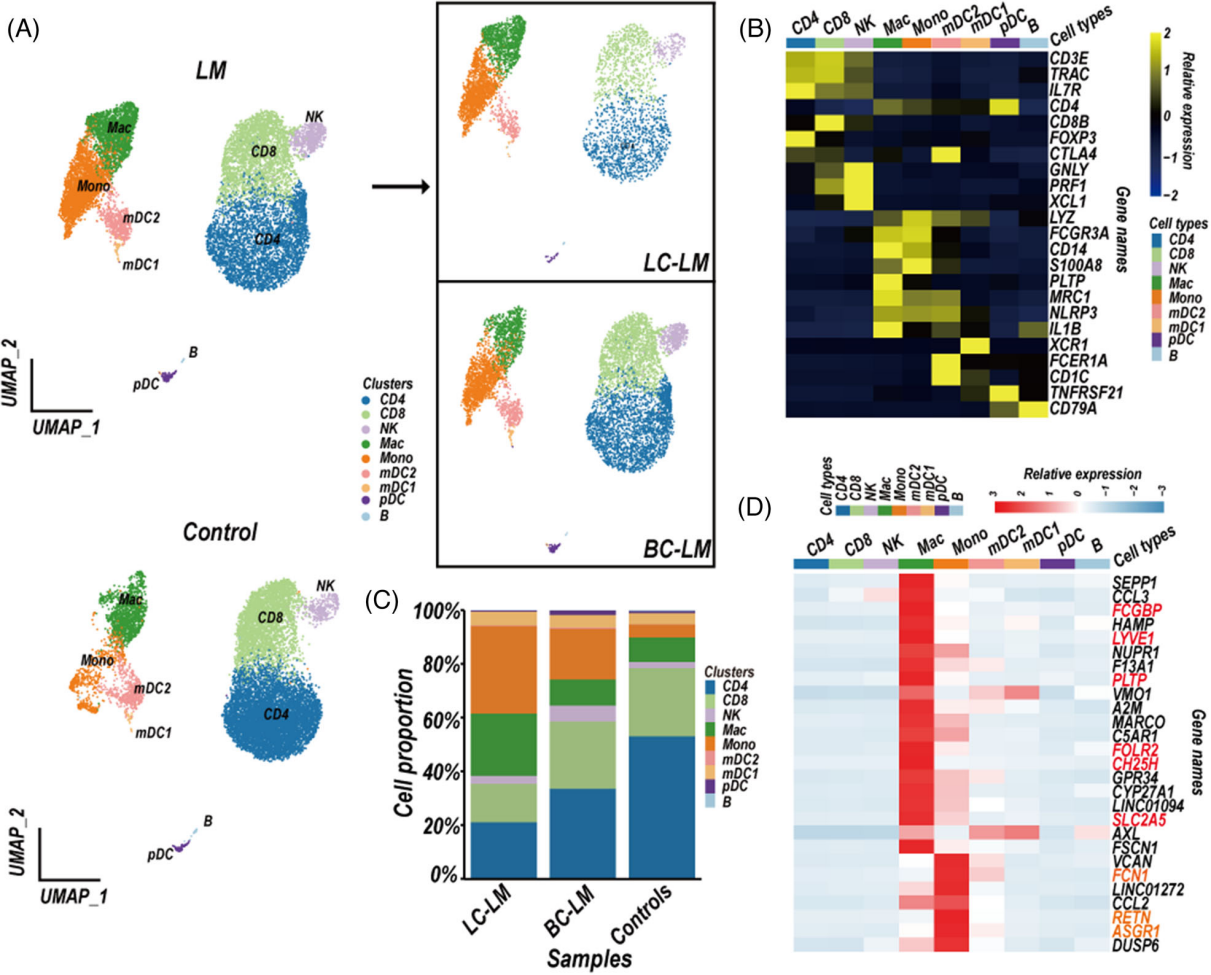
Leptomeningeal metastases (LM) predominantly alter cerebrospinal fluid (CSF) immune cell composition. (A) Uniform Manifold Approximation and Projection (UMAP) plot showing profiled cells from CSF samples of five LM patients (LM, top left), four controls (control, bottom left), two LC‐LM patients or three BC‐LM patients (top right). (B) Canonical marker gene expression was depicted in each cluster. Colour encodes the average gene expression level. (C) The proportion of each cluster in CSF samples of BC‐LM patients, LC‐LM patients, and control samples. (D) Heatmap of the signature genes of macrophages and monocytes. Log_2_Foldchange > 1, adjusted *P*‐value <  0.01, gene expressed in > 25% of cells in one cluster (marked as PCT, percentage of cells) and PCT < 5% in other clusters. Cluster key: *pDC*, plasmacytoid dendritic cells; *mDC1*, myeloid DC type 1; *mDC2*, myeloid DC type 2; *Mono*, monocytes; *Mac*, macrophages; *CD8*, CD8^+^ T cells; *CD4*, CD4^+^ T cells; *NK*, natural killer cells; *B*, B cells. Sample ID: patient CSF samples, three LM patients of breast cancer (*BC‐LM‐B, BC‐LM‐C, BC‐LM‐E*), two LM patients of lung cancer (*LC‐LM‐A, LC‐LM‐D*), GSE150681; Control CSF samples, Control 1–4, GSE138266

Immune components have been reported to exert crucial influence on tumorigenesis and progression,^19^ we first sought to identify the characteristics of CSF immune cells in BC‐LM and LC‐LM patients compared to patients with IIH as controls. We obtained in total 26,274 CSF single‐cell transcriptomes data (four controls vs. five LM) and classified them into 9 final cell clusters (Figures 1A and S1A, Table S1).

T cells (CD3E and TRAC) were divided into CD4+ T cells (IL7R and CD4) and CD8+ T cells (CD8B) (Figures 1B and S1B). Myeloid lineage cells (LYZ) were separated into mDC type 2 (mDC2; FCER1A and CD1C), mDC type 1 (mDC1; XCR1), monocyte cell cluster (Mono; FCGR3A/CD16 and CD14) and macrophage cluster (Mac; MRC1, PLTP, and IL1B) (Figures 1B and S1B). Additional clusters represented natural killer (NK) cell clusters (GNLY, PRF1 and XCL1), plasmacytoid dendritic cells (pDC; TNFRSF21), and B cell cluster (CD79A) (Figures 1B and S1B). We next analysed the difference of cell composition in LM CSF compared to controls. As expected, the monocyte and macrophage clusters showed higher proportion in LM than these in controls (Figures 1C and S1C). We intended to characterise the signature genes of the monocyte and macrophage clusters (Table S2). We applied the FindAllMarkers function in R package Seurat (4.0.4) to select signature genes in different cell clusters according to the statistical threshold (log2FoldChange   ≥  1, adjusted *P*‐value <  0.01). Signature genes need to be expressed in > 25% of cells in a cell cluster and < 5% in other immune cell clusters. As expected, the macrophage cluster expressed markers of microglia (TREM2 and TMEM119^20^), and CNS border‐associated macrophages (CH25H^21^) (Figure S1D). The genes *CH25H, LYVE1, FSCN1, FOLR2, PLTP, SLC2A5 and FCGBP* were candidate signature genes in CSF macrophage cluster, whereas *RETN, ASGR1 and FCN1* have the possibility to serve as signature genes of CSF monocyte cluster (Figures 1D and S1D).

### 3.2 Macrophages in CSF of LM patients show M2‐polarised phenotype

**FIGURE 2:**
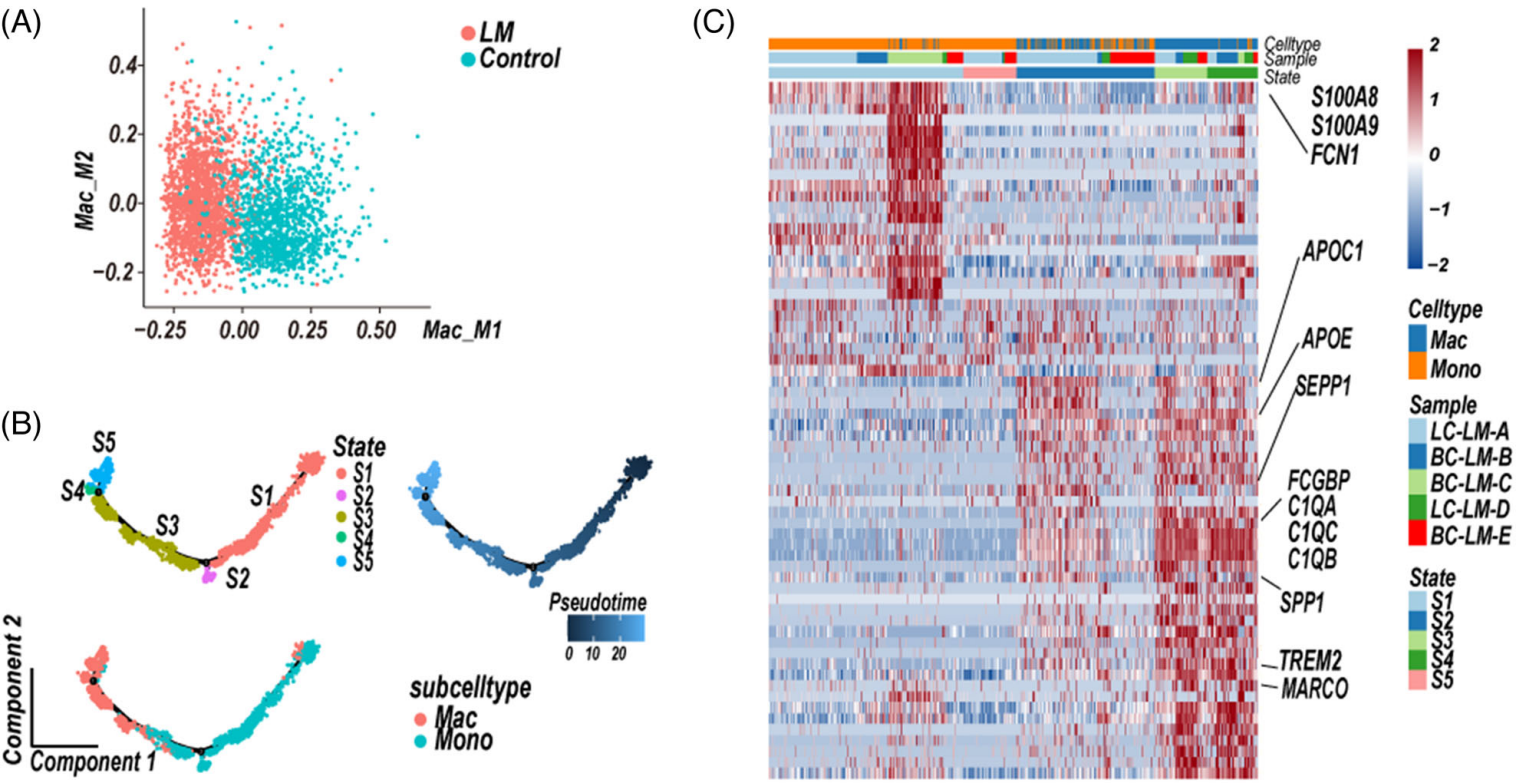
Macrophages in CSF of leptomeningeal metastases (LM) patients show M2‐polarised phenotype. (A) Scatterplots of normalised scores of the M1‐subtype (Mac_M1) or M2‐subtype (Mac_M2) signature genes in each macrophage (dot) from CSF samples of LM (red) and control (blue) samples. (B) Unsupervised trajectory of monocytes and macrophages state transitions in LM CSF samples. The branched trajectory was coloured by cell states, cell subsets, and pseudo‐time. (C) Relative expression map of upregulated genes specific to cell states. Selected genes in each state are indicated on the right

Myeloid cells play a critical role in tumour inflammation regulation. Tumour‐associated macrophages (TAMs) are commonly characterised as M2‐polarised phenotype macrophages, which contribute to tumour aggressiveness.^22^ M2‐subtype macrophages, the alternative activated macrophages, showed pro‐tumorigenic ability, whereas M1‐subtype macrophages, the conventional classic activated macrophages, exhibited antitumorigenic activity.^23^ To understand the polarisation of macrophages in LM CSF samples, we analysed the expression of M1‐subtype and M2‐subtype signature genes in macrophage cluster.^24^ Interestingly, the macrophages in LM CSF samples were more likely to show M2‐subtype signature, whereas the control macrophages had similar expression level of both M1‐subtype and M2‐subtype signature genes (Figure 2A).

An unsupervised trajectory analysis was conducted to understand the transcriptional transition from monocytes to macrophages in LM CSF samples.^25^ The pseudo‐time axis in LM CSF samples showed a series of transitions from pro‐inflammatory monocytes to M2‐subtype macrophages showing anti‐inflammatory signatures (Figure 2A,B, Table S3).^26^, ^27^ Whereas in control CSF sample, unsupervised trajectory of monocytes and macrophages state transitions eventually showed macrophages at state 4 (S4) were enriched both pro‐ or anti‐inflammatory signatures genes (Figures S2A,B, Table S3). Therefore, we further performed an unsupervised trajectory analysis of monocytes and macrophages at state 1 (S1) and state 4 (S4). The pseudo‐time axis reached a branching point, at which macrophages were enriched either in pro‐ (state 9, S9) or anti‐inflammatory signatures (state 10, S10), respectively (Figure S2C,D, Table S3). Compared to M2‐subtype macrophages, M1‐subtype macrophages (Figure S2C,D, Table S3) in controls were enriched expression of inflammatory factors (*CCL3, CCL4, CCL4L2, CCL3L3 CCL8, CXCL8*).

### 3.3 Regulatory T cells increase in CSF of LM patients

**FIGURE 3:**
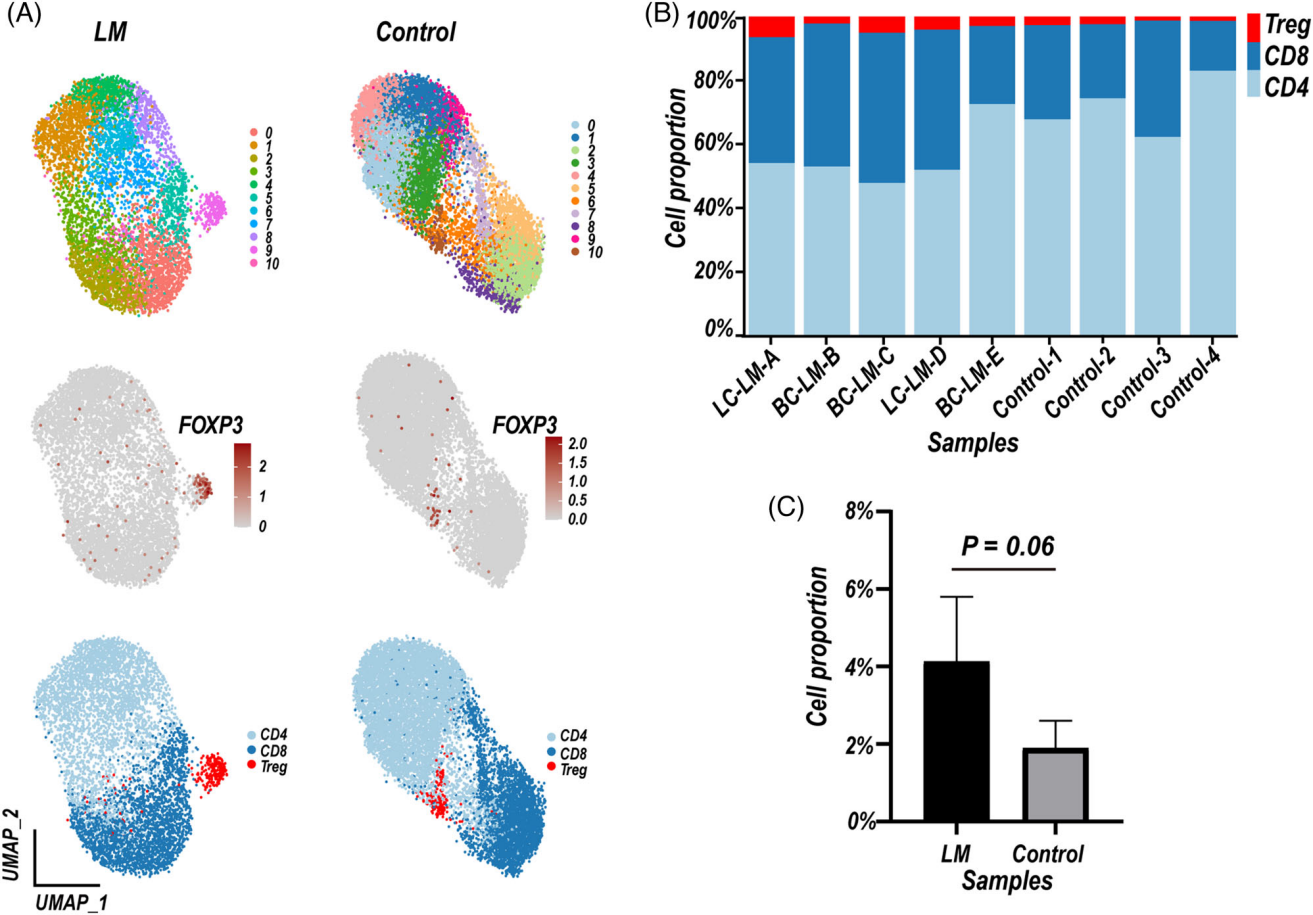
Characteristics of T cells in cerebrospinal fluid (CSF) samples of leptomeningeal metastases (LM) patients and controls. (A) Uniform Manifold Approximation and Projection (UMAP) plot of T cells, colour‐coded by clusters and cell subsets as indicated in Control (up) and LM (down) samples. (B) Relative proportion of T cell subsets in each sample. Sample ID: Patient CSF samples, three LM patients of breast cancer (BC‐LM‐B, BC‐LM‐C, BC‐LM‐E), two LM patients of lung cancer (LC‐LM‐A, LC‐LM‐D), GSE150681; Control CSF samples, Control 1–4, GSE138266. (C) Bar plot of the percentage of regulatory T cells (Tregs) subset in LM and control samples. Data represent mean ± SD, LM, 4.122% ± 1.498%, Control, 1.889% ± 0.615%. *P*‐value (*P*) was calculated using the Mann–Whitney test, *P* = 0.0635; LM, *n* = 5 samples, Control, *n* = 4 samples. T cell subsets key: CD4, CD4+ T cells, CD8, CD8+ T cells, Tregs, regulatory T cells

T lymphocytes play central roles in mediating anti‐tumour immunity and immune therapies. We sub‐clustered T cells based on marker gene expression. CD8+ T cells (*CD8A and CD8B*) displayed cytotoxic (*GZMA, GNLY, GZMB, GZMK, IFNG, NKG7*) or exhausted state (*LAG3, TIGIT, PDCD1, HAVCR2*) (Figures 3A,B and S3), CD4+ T cells (*CD4 and IL7R*) mainly showed naïve state (Figures 3A,B and S3). In addition, the upregulated proportion of regulatory T cells (Tregs, *FOXP3, IL2RA, TNFRSF4, TNFRSF18*) in LM compared to controls (control vs. LM, mean proportion 4.040% vs. 1.825%), delivered a direction of tumour immunosuppression (Figures 3A–C and S3).

### 3.4 The characteristics of CSF‐CTCs of breast cancer LM

**FIGURE 4:**
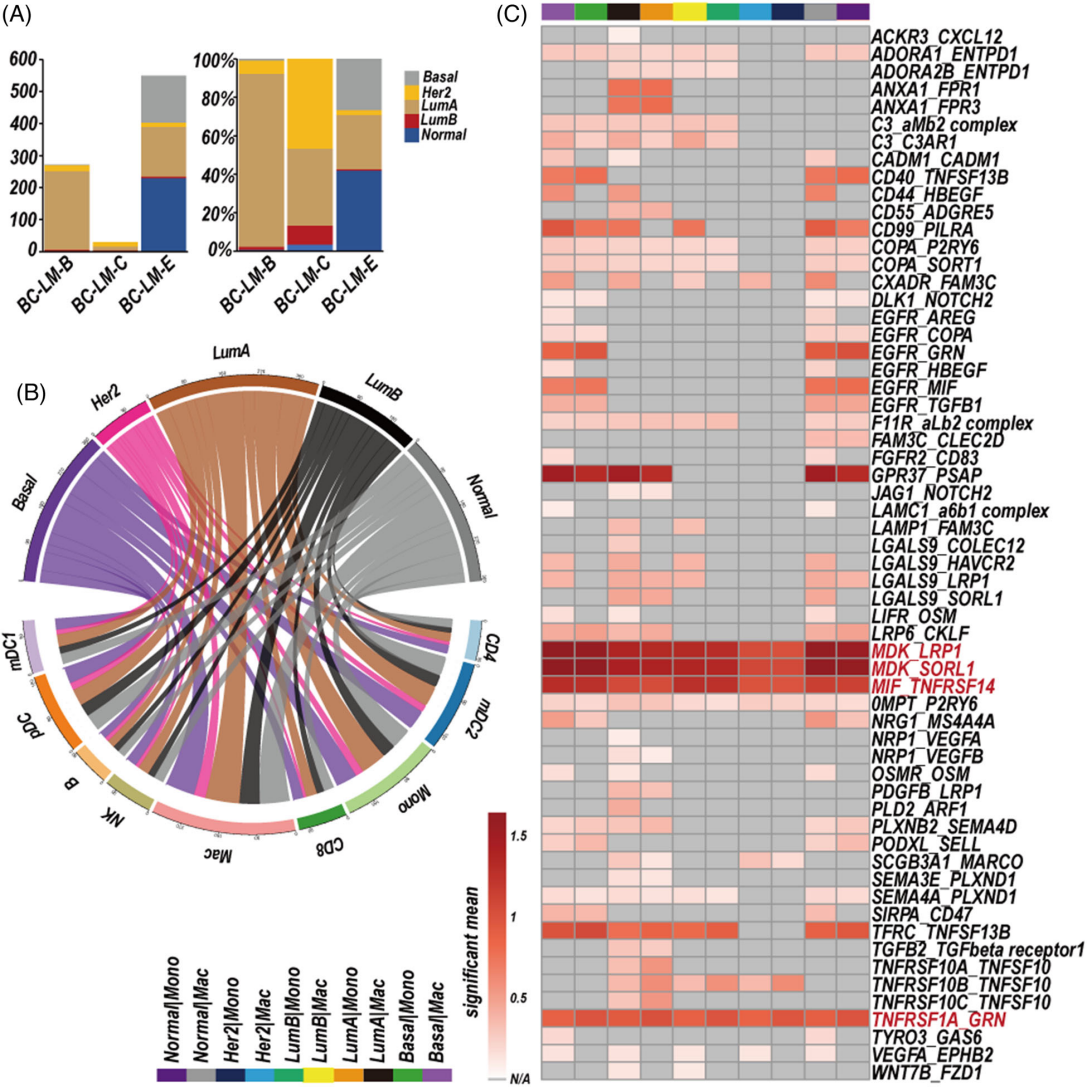
Cell–cell interaction network between cerebrospinal fluid circulating tumour cells (CSF‐CTCs) and immune cells. (A) Cell number and relative proportion of five subtypes of CSF‐CTCs from three leptomeningeal metastases (LM) patients of breast cancer (BC‐LM) based on PAM50 model (BC‐LM‐B, BC‐LM‐C, BC‐LM‐E). (B) Cell–cell interaction network between five subtype‐specific CSF‐CTCs and different immune cell types. The line thickness represents the number of significant ligand–receptor interactions in two cell types. *P*‐value < 0.05 was considered as significant interaction. (C) Heatmap depicting significant mean of interactions between five subtype‐specific CSF‐CTCs and macrophages (Basal|Mac, LumA|Mac, LumB|Mac, Her2|Mac, Normal|Mac) or monocytes (Basal|Mon, LumA|Mon, LumB|Mon, Her2|Mon, Normal|Mon). Subtype key: Normal, normal‐like; Basal, basal‐like; Her2+, Her2 positive; LumA, Luminal A; LumB, Luminal B. Mac, macrophages; Mon, monocytes. N/A, Not applicable

CSF‐CTCs are potential biomarkers for BC diagnosis and prognosis.^28^ Our previous study had showed the characteristics of LC CSF‐CTCs, here we focused on BC CSF‐CTCs. We identified BC‐LM CSF‐CTCs by epithelial cell markers (*EPCAM, CDH1, KRT18 and KRT8*) expression and a higher variable of copy number variations (CNVs) than normal immune cells (Figure S4). First, we performed the PAM50 model to classify the BC‐LM CSF‐CTCs into five molecular subtypes according to 50 PAM50 signatures genes.^10^ 852 CSF‐CTCs were defined as normal‐like (normal, 148 cells), basal‐like (basal, 233 cells), Her2 positive (Her2, 11 cells), Luminal A (LumA, 413 cells), and Luminal B (LumB, 47 cells) (Figures 4A, S5, Table S4).

Second, the python CellPhoneDB package^11^ was applied to investigate the cell–cell communication, ligand–receptor interaction with *P*‐value < 0.05 was thought to be significant. The macrophages and five subtype‐specific CSF‐CTCs had largest number of ligand–receptor interactions (Figure 4B). The MDK‐SORL1 and MDK‐LRP1 ligand–receptor pairs were significantly interacted in the five subtype‐specific CSF‐CTCs and macrophages (monocytes) (*P*‐value < 0.05; Figure 4C, Table S5). Increased MDK in tumour cells promoted immunosuppressive differentiation of tumour‐infiltrating macrophages by an interaction with its receptor LRP1 in ErbB pathway‐mutated gallbladder cancer.^29^ MDK has potential to regulate macrophages differentiation in TME of LM patients.

**FIGURE 5:**
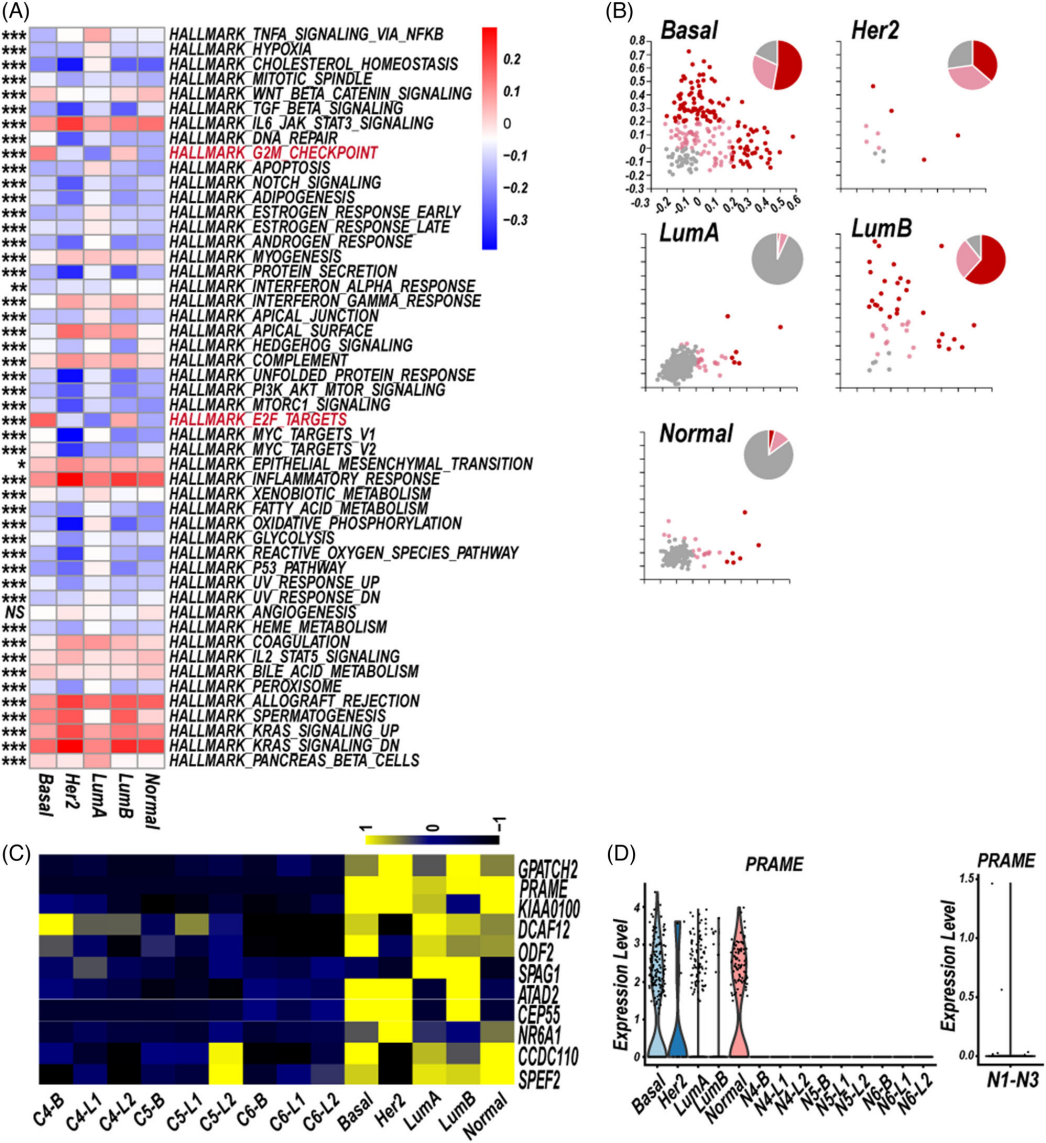
The heterogeneity of five subtype‐specific cerebrospinal fluid circulating tumour cells (CSF‐CTCs). (A) Differences of the enrichment of the hallmark gene sets across the five subtype‐specific CSF‐CTCs. The colours are encoded by the mean values of the gene set variation analysis (GSVA) enrichment scores in the five molecular subtypes (one‐way ANOVA, ***P‐value < 0.001; **P‐value < 0.01; *P‐value < 0.05; NS, *P*‐value > = 0.05). (B) Scatterplots of the cell cycle state of every CSF‐CTC (circle) based on the expression of G1/S (*x*‐axis) and G2/M (*y*‐axis) gene sets in the five subtypes. Cells are coloured by cell‐cycle states. Score > 0.2, cycling cells (red); 0 < score ≤ 0.2, intermediate state (pink); score ≤ 0, noncycling cells (gray). Pie chart showing the relative proportion of cell‐cycle state of five subtype‐specific CSF‐CTCs. (C) Heat map showing the relative expression of 11 CTAs in five subtype‐specific CSF‐CTCs, and normal breast epithelial cells of one basal cluster (B) and two luminal clusters (L1 and L2) from three normal samples (N4, N5, N6, GSE113196). (D) Violin plots of *PRAME* expression in 12 clusters (C) and normal breast epithelial cells of three other normal samples (N1–N3, GSE113099, GSE113127, and GSE113198). Subtype key: Normal, normal‐like; Basal, basal‐like; Her2, Her2 positive; LumA, Luminal A; LumB, Luminal B

Third, we analysed the heterogeneity of BC‐LM CSF‐CTCs of the five different subtypes, from the aspects of gene set enrichment, CTAs expression and cell‐cycle state. A GSVA was performed to identify activity of 50 hallmark gene sets in five subtype‐specific CSF‐CTCs, which showed intratumoral heterogeneity. The basal and LumB subpopulations showed significant enrichment of the G2/M checkpoint and E2F targets (Figure 5A).

Gene signatures of S or G2/M phases were performed to characterise proliferation state of five subtype‐specific CSF‐CTCs. Cycling CSF‐CTCs were enriched for cell‐cycle phase‐specific signatures, distinguishing from noncycling cells (Figure 5B). The basal and LumB subpopulations had higher proportion of cycling CSF‐CTCs than other subtype‐like subpopulations (Figure 5B).

Previous studies have shown the CTAs characteristics of CSF malignant cells of LC^3^ and central nervous system lymphoma.^4^ Here, we focused on CTAs expression (http://www.cta.lncc.br/modelo.php) in the five subtype‐specific CSF‐CTCs. BC CSF‐CTCs had higher expression of 11 CTAs (Figure 5C, Table S6; PCT in CSF‐CTCs > 0.05), compared to normal breast epithelial cells of basal clusters and luminal clusters from three samples (GSE113196, Normal sample 4–6, N4–N6).^12^ Interestingly, the CTA PRAME (36.38%, 310/852) were greatly restricted to be expressed in the five subtype‐specific CSF‐CTCs, whereas little to no expression was in normal breast epithelial cells (Figure 5D), which could be served as potential immunotherapy targets.

### 3.5 Gene regulatory networks of five subtype‐specific CSF‐CTCs of BC‐LM

**FIGURE 6:**
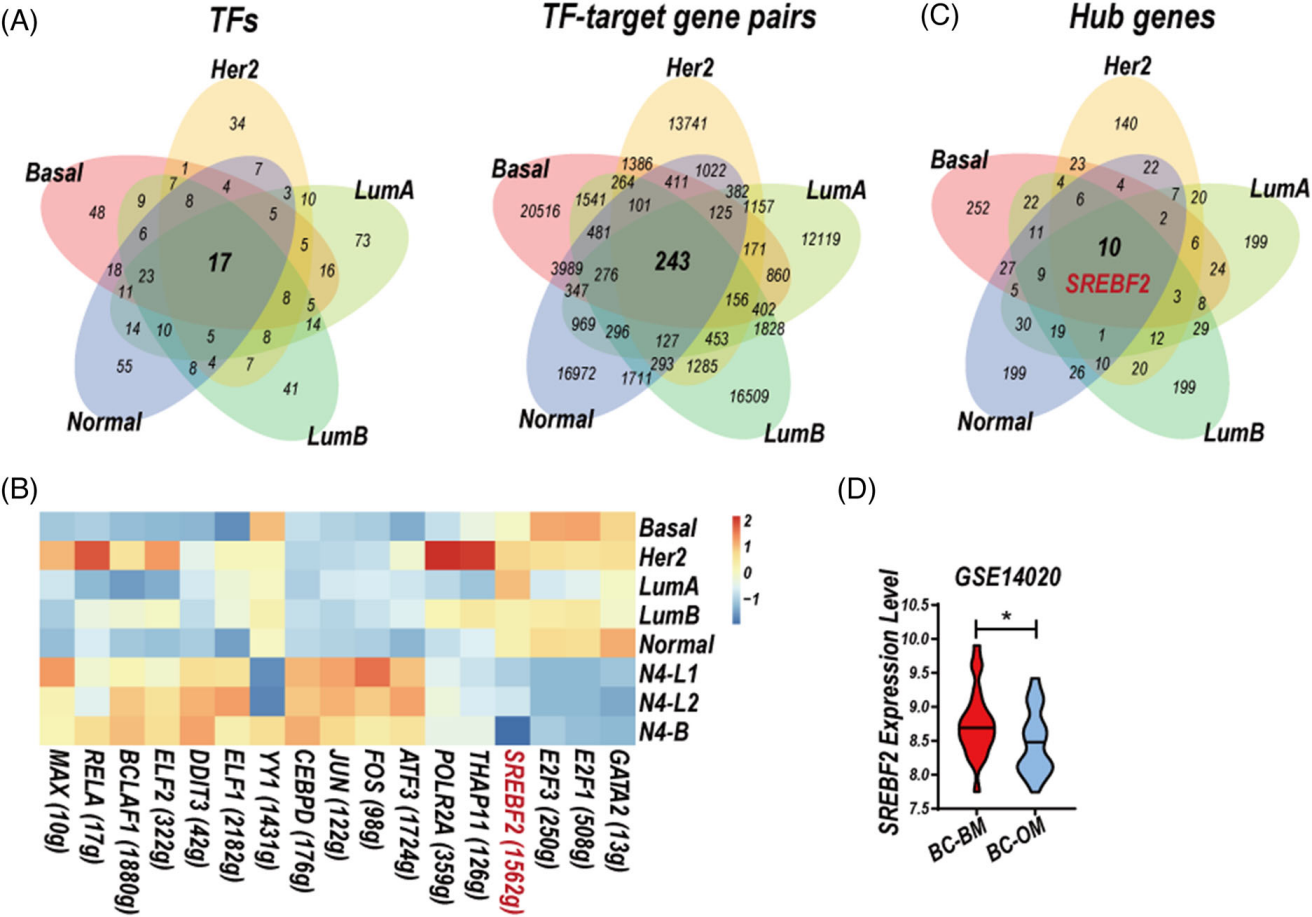
Gene regulatory networks (GRNs) of five subtype‐specific cerebrospinal fluid circulating tumour cells (CSF‐CTCs) and normal epithelial cells. (A) Venn diagram of regulators (TFs, left) and co‐expressed TF–target gene pairs (right) of the five subtype‐specific GRNs. (B) Heatmap showing the average activity of 17 TFs regulation calculated by AUCell R‐package in five subtype‐specific CSF‐CTCs, and normal breast epithelial cells of one basal cluster (N4‐B) and two Luminal clusters (N4‐L1 and N4‐L2) from one normal sample (N4, GSE113197). (C) Venn diagram of hub genes in the five molecular subtype‐specific GRNs. (D) Violin plot of *SREBF2* expression in breast cancer metastatic tumour tissues of brain metastases (BC‐BM) and other metastases (BC‐OM), GSE14020. *P‐value < 0.05, two‐sided Student's *t*‐test

The GRNs construction of three clusters of normal epithelial cells^12^ and five subtype‐specific CSF‐CTCs were performed by R package SCENIC.^17^ 17 TFs and 243 TF–target gene pairs were conserved in the GRNs of five subtype‐specific CSF‐CTCs (Figure 6A). We performed AUCell in R‐package SCENIC to identify cells with active TF regulation. Among the 17 TFs, the activity of SREBF2 (sterol regulatory element binding transcription factor 2) was upregulated in the GRNs of five subtype‐specific CSF‐CTCs compared to three clusters of normal epithelial cells (Figure 6B, Table S7). In addition, we identified the hub gene in the 5 GRNs by CytoHubba in Cytoscape.^18^ The hub genes in five subtype‐specific CSF‐CTCs were greatly diverse, 10 critical genes were activated in the five GRNs, including the important TF SREBP2 (Figure 6C).

TFs sterol regulatory element‐binding proteins (SREBPs) family is necessary for the synthesis of fatty acids and cholesterol.^30^ Activation of SREBP1 and SREBP2 has been reported to promote BC proliferation.^31^ Moreover, a feedback loop of CtBP‐ZEB1‐SREBF2 contributed to BC metastases through the repression of cholesterol and activation of TGF‐β signalling.^32^ SREBF2 expression was higher in BC metastatic tumour tissues of brain metastases (BC‐BM) compared to other metastases (BC‐OM, Figure 6D).

### 3.6 The characteristics of cerebrospinal fluid cells derived from one breast cancer leptomeningeal metastases patient (BC‐LM‐F) validating discoveries

**FIGURE 7:**
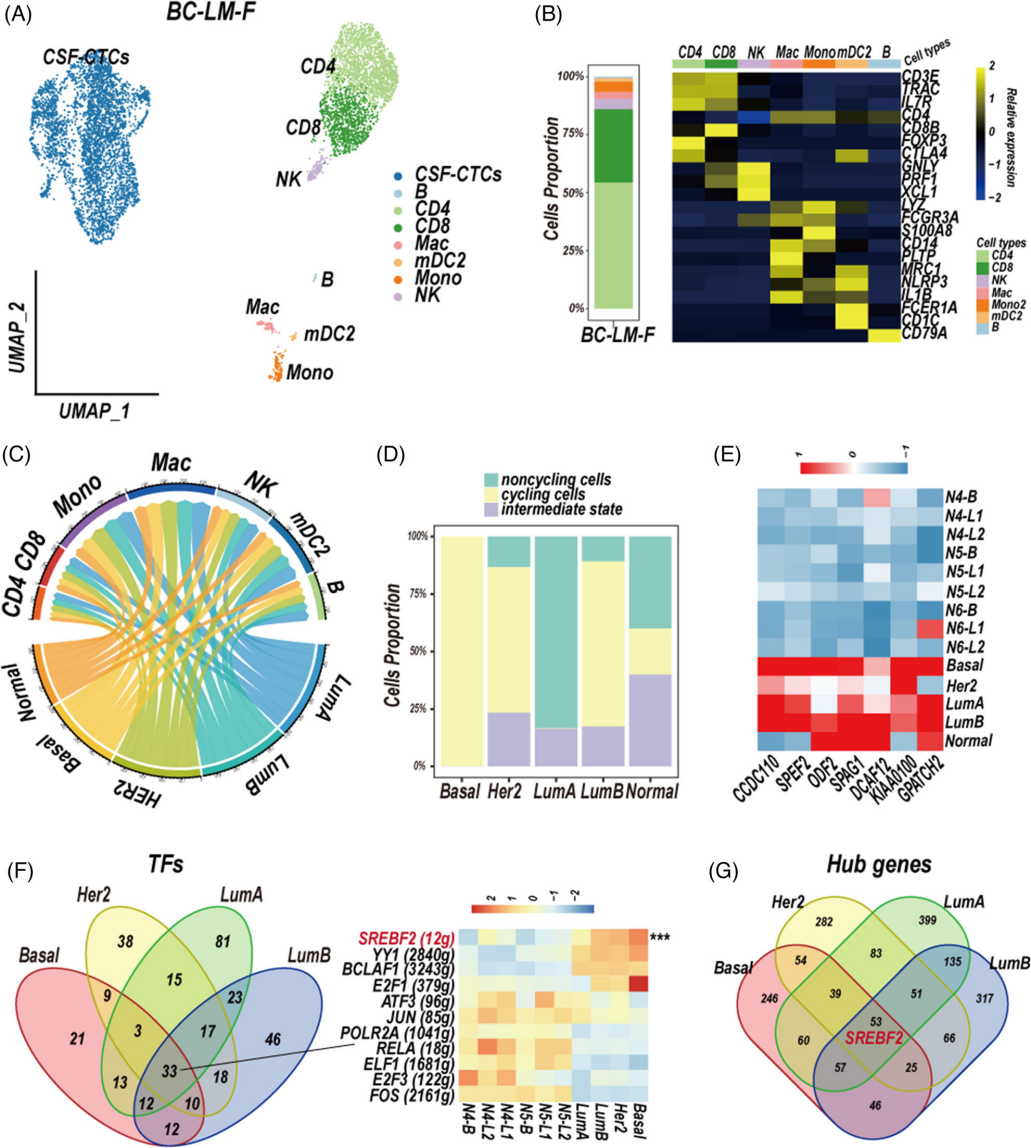
The characteristics of cerebrospinal fluid (CSF) cells derived from one breast cancer leptomeningeal metastases patient (BC‐LM‐F). (A) Uniform Manifold Approximation and Projection (UMAP) plot showing profiled cells from BC‐LM‐F CSF sample. (B) The proportion (left) and the markers expression (right) of each cluster in BC‐LM‐F CSF sample. Cluster key was same as that in Figure 1. (C) Cell–cell interaction network between five subtype‐specific CSF‐CTCs and different immune cell types in BC‐LM‐F CSF sample. The line thickness represents the number of significant ligand–receptor interactions in two cell types. *P*‐value < 0.05 was considered as significant interaction. (D) Histogram showing the relative proportion of cell‐cycle state of CSF‐CTCs based on the expression of G1/S (*x* axis) and G2/M (*y* axis) gene sets in the five subtype‐specific CSF‐CTCs. Score > 0.2, cycling cells (yellow); 0 < score ≤ 0.2, intermediate state (purple); score ≤ 0, noncycling cells (green). (E) Heat map showing the relative expression of 7 CTAs in five subtype‐specific CSF‐CTCs, and normal breast epithelial cells. (F) Venn diagram (left) showing regulators (TFs) of the gene regulatory networks (GRNs), heatmap (right) showing the average activity of 11 TFs regulation calculated by AUCell R‐package in four subtype‐specific CSF‐CTCs derived from BC‐LM‐F CSF sample and normal breast epithelial cells. Normal breast epithelial cells of one basal cluster (B) and two luminal clusters (L1 and L2) were from normal samples (N4, N5, N6, GSE113196). (G) Venn diagram of hub genes in the four subtype‐specific GRNs

In order to validate our discoveries, we performed 10× single‐cell RNA sequencing on the CSF cells derived from a BC leptomeningeal metastases patient (BC‐LM‐F). We removed cells which had less than 300 genes and 1000 counts, more than 20% mitochondrial‐gene counts, and computed doublet detection by DoubletFinder (https://github.com/ddiez/DoubletFinder). Six thousand nine hundred forty‐four CSF single‐cell transcriptomes data in total were obtained and classified into eight final cell clusters, no obvious pDC cluster and mDC1 cluster were discovered (Figures 7A,B and S8A, Table S1; cell counts: CSF‐CTCs, 4004; B, 18; CD4, 1602; CD8, 928; NK, 128; Mac, 92; Mono, 124; mDC2, 48). CSF‐CTCs were identified by epithelial markers and higher variable of CNVs than normal immune cells (Figure S8B).

We first focused on the immune cells. Macrophages signature genes CH25H, LYVE1, FSCN1, FOLR2, PLTP, SLC2A5 and FCGBP, and monocytes signature genes RETN, ASGR1 and FCN1 were conformed in the BC‐LM‐F CSF sample (Figure S9, Table S2). An unsupervised trajectory analysis showed a series of transitions from pro‐inflammatory monocytes to anti‐inflammatory M2‐subtype macrophages (Figure S10A,B). We sub‐clustered T cells and discovered the proportion of regulatory T cells in BC‐LM‐F CSF sample was 2.192% (CD4, 1495 cells; CD8, 914 cells, Treg, 54 cells; Figure S10C).

Then, we performed the PAM50 model to classify the CSF‐CTCs of BC‐LM‐F into five molecular subtypes, most CSF‐CTCs are LumA subtype (Table S4; LumA, 3313 cells, LumB, 46, HER2, 30, Basal, 11, Normal, 5). Cell–cell communication analysis by the python CellPhoneDB package showed macrophages and five subtype‐specific CSF‐CTCs had largest number of ligand–receptor interactions (Figure 7C, Table S5). The MDK‐SORL1, MDK‐LRP1, MIF‐TNFRSF14, TNFRSF1A‐GRN ligand–receptor pairs, and CD74‐MIF, CD74‐COPA, CD74‐APP, GRN‐SORT1 pairs were significantly interacted between five subtype‐specific CSF‐CTCs and macrophage/monocytes communication (Figure S11).

The heterogeneity of five‐subtype CSF‐CTCs of BC‐LM‐F patient was also showed on gene set enrichment, CTAs expression and cell‐cycle state. The basal and LumB subpopulations showed significant enrichment of the G2/M checkpoint and E2F targets (Figure S12A), and had higher proportion of cycling CSF‐CTCs than other subtype‐like subpopulations (Figure 7D). We focused on the 11 CTAs (Figure 5C, Table S6), and discovered seven CTAs were also upregulated in CSF‐CTCs of BC‐LM‐F patient compared to normal epithelial cells (Figure 7E). However, the CTA PRAME was not expressed in CSF‐CTCs of BC‐LM‐F patient. The GRNs construction showed 33 TFs and 1154 TF‐target gene pairs (Figures 7F and S12B) were activated in four subtype‐specific CSF‐CTCs (LumA, LumB, HER2, Basal). Here, the basal‐like CSF‐CTCs were not analysed for the few cells (five cells). We paid attention to the 17 TFs (Figure 6A), 11 TFs were also conserved in the GRNs of four subtype‐specific CSF‐CTCs from the BC‐LM‐F sample (Figure 7F). Compared to normal epithelial cells, the activation of SREBF2 was also upregulated in the GRNs of CSF‐CTCs, which also served as hub gene (Figure 7F,G).

## 4 DISCUSSION

LM, referring to the spread of cancer cells into the CSF, is typically fatal within months.^33^ The leptomeningeal space, isolated by the blood–CSF–barrier, is hypoxic and contains sparse amounts of metabolic intermediates and micronutrients.^34^ Interests always abound in the mechanisms how cancer cells in CSF cope with oppressive metabolic constraints? Cancer cells of BC‐LM and LC‐LM make use of iron collection system LCN2/SLC22A17 to outcompete the macrophages, the major iron‐utilising cells, for sparse environmental iron, which promotes cancer cells growth and evades immune responses.^8^ Cancer cells of BC‐LM and LC‐LM also disrupt the blood–CSF barrier by deriving C3 to activate the C3a receptor in the choroid plexus epithelium to allow plasma amphiregulin and other mitogens entering the CSF, which also promotes cancer cells growth in CSF.^35^ In addition, single‐cell transcriptomic techniques were applied to focus on the characteristics of CSF‐CTCs of LC‐LM, which showed great heterogeneity.^3^ However, the comprehensive analysis of CSF TME of LM have not been performed.

In this study, we concentrated on the CSF TME of BC‐LM and LC‐LM patients, and the characteristics of BC‐LM CSF‐CTCs based on the scRNA‐seq data. We identified nine different immune cell types, and revealed monocytes and macrophages enriched in CSF of LM patients (Figure 1C, Table S1). M2‐polarised phenotype macrophages in TME are a major component contributing to tumour immunosuppression. Single‐cell RNA sequencing data have revealed macrophages with an M2 phenotype displayed immunosuppressive characteristics in primary BC^36^ and triple‐negative BC,^37^, ^38^ as well as metastatic lung adenocarcinoma.^27^ The macrophages in CSF samples of BC‐LM and LC‐LM patients also showed M2‐subtype signature and enriched KEGG fatty acid metabolism pathway (NES = 1.788, FDR *q*‐value = 0.003, GSEA analysis), which providing a crucial energy source for M2 polarization.^39^

The paracrine interactions between malignant cells and TME immune cells promote the immune surveillance escape and tumour progression. Interactions between myeloid cells (monocytes, macrophages and mDC2) were the most prominent in the network of immune cells (Figure S6). The macrophages and CSF‐CTCs had largest number of ligand–receptor interactions in the TME (Figure 4B). In addition to the MDK‐SORL1 and MDK‐LRP1 ligand–receptor pairs, the MIF‐TNFRSF14, TNFRSF1A‐GRN, CD74‐MIF, CD74‐COPA, CD74‐APP, and GRN‐SORT1 pairs also showed great importance in the macrophage/monocyte‐CTCs communication (Figures 4C and S7, Table S5).

BC CSF‐CTCs had great heterogeneity shown on the cell‐cycle state and CTAs expression (Figure 5B,C), same as LC CSF‐CTCs^3^ and CSF‐DLBCs.^4^ CSF‐CTCs in basal‐like subtype, which always has higher probability of metastases and worse prognosis than other subtypes, showed highest proliferation ability (Figure 5A,B). The CTAs expression were various in the five subtype‐specific CSF‐CTCs, and the CTA PRAME restricted to CSF‐CTCs are the potential therapeutic target (Figure 5C,D, Table S6). The GRNs of the five subtype‐specific CSF‐CTCs also showed great heterogeneity. The hub gene transcription factor SREBF2 was activated in the five subtype‐specific CTCs (Figure 6C, Table S7).

We enrolled one BC‐LM patient to help generate a solid conclusion and 6944 CSF single‐cell transcriptomes data in total were obtained for analysis (Figure 7A,B). The TME analysis showed M2‐polarised phenotype macrophages and the largest number of ligand–receptor interactions between macrophages and CSF‐CTCs (Figures 7C, S10 and S11). In addition, the great heterogeneity of CSF‐CTCs was discovered and the GRNs of CSF‐CTCs also showed the importance of SREBF2 (Figures 7D–F and S12).

CTCs in blood of BCs have been reported into two subpopulations: epithelial (mesenchymal‐to‐epithelial transition, or MET) cells and mesenchymal (epithelial‐to‐mesenchymal transition, or EMT) cells.^15^ We also preformed EMT analysis on various CTCs. Blood‐CTCs sequenced by Hydro‐SEquation (Blood‐CTCs‐Hydro, 693 cells),^15^ by Smart‐seq2 method (blood‐CTCs‐Smart, 35 cells),^13^ and by 10× genomics scRNA‐seq method (blood‐CTCs‐10×, 75 cells)^14^ showed both MET and EMT characteristics, whereas CSF‐CTCs of BC‐LM‐B, BC‐LM‐C, BC‐LM‐E, BC‐LM‐F samples were only in the MET state (Figure S13A,B). In addition, blood CTCs and CSF‐CTCs are excellent materials for studying the process of BC‐LM. We intend to analyse how rare “speculative cells” in blood to develop BC‐LM by identifying the transcriptome difference between CSF‐CTCs and blood‐CTCs. We performed the DEGs analysis between blood‐CTCs and CSF‐CTCs of four BC‐LM patients, 8 genes (SMIM22, MDK, NPDC1, PLPP2, METRN, SCD, BICDL2, NR2F2) were significantly upregulated both in CSF‐CTCs compared to blood‐CTCs (adjusted *P*‐value < 0.05, FoldChange > 1.5, Figure S13C,D), deserving attention and further study. MDK is a heparin‐binding growth factor overexpressed in tissues of BC brain metastases (Figure S14A), CSF‐CTCs (Figure S14C) and BC tissues (Figure S14D,E), whereas there was no obvious difference of MDK expression in the five subtype‐specific BC tissues (Figure S14B), we have discovered MDK‐SORL1 and MDK‐LRP1 ligand–receptor pairs were significantly interacted in the five subtype‐specific CSF‐CTCs and macrophages (monocytes) (Figures 4C and S11). Interestingly, in control CSF samples, immune cells have little to no expression of MDK, whereas in CSF samples of BC‐LM patients, the expression of secreted protein MDK was greatly upregulated, no matter in CSF‐CTCs or CSF immune cells (Figure S14F). These results showed that MDK might be a potential drug target and diagnostic marker for BC‐LM. However, it needs be pointed out that the differences analysis between CSF‐CTCs and blood‐CTCs were based on different dataset and different patients, therefore further experimental verification needs to be performed to make our results more reliable.

In summary, our study is the first one to define the single‐cell transcriptome characteristics of CSF TME in LM patients of BC and LC. The emergence of M2‐subtype macrophages and regulatory T cells in LM confirmed the direction of tumour immunosuppression. The communication network in CSF TME showed most significant interactions between BC five subtype‐specific CSF‐CTCs and macrophages/monocytes, especially the interactions of MDK‐SORL1 and MDK‐LRP1 ligand–receptor pairs. The great heterogeneities of BC CSF‐CTCs were manifested in cell proliferation, CTAs expression and GRNs. GRNs revealed that SREBF2 was critical for the BC‐LM progression. Our study provided an important reference point for CSF microenvironmental research of LM and revealed transcriptional plasticity of BC CSF‐CTCs, which facilitated the understanding of LM mechanism research. Further, we intend to enlarge specimen to further support and enrich research results. It is interesting to analyse the association between survival and CSF immune cell composition.

## CONFLICT OF INTEREST

The authors declare that there is no conflict of interest that could be perceived as prejudicing the impartiality of the research reported.

## Supporting information

Supporting Material

Supporting Material

Supporting Material

Supporting Material

Supporting Material

Supporting Material

Supporting Material
