# Supplementary material for: Single‐cell RNA sequencing reveals the characteristics of cerebrospinal fluid tumour environment in breast cancer and lung cancer leptomeningeal metastases
Source: Clin Transl Med. 2022 Jun 9;12(6):e885. doi: 10.1002/ctm2.885 (PMC9178395; doi:10.1002/ctm2.885)
Supplement: Supplementary file 1 — Supporting Material [file CTM2-12-e885-s002.docx]

FIGURE S1 Cerebrospinal fluid (CSF) immune cell composition.

(A) UMAP plot of 26,274 profiled cells from nine CSF samples of five leptomeningeal metastases (LM) patients and four controls. (B) UMAP showing the expression of cluster marker genes (*Figure S1A*). (C) The proportion and cell number of each cluster in nine CSF samples. (D) Feature plots demonstrating the expression of signature genes of monocyte or macrophage cluster on the UMAP plot (*Figure S1A*). Scaled expression levels are depicted using a red gradient (grey denotes lack of expression). Cluster key: pDC, plasmacytoid dendritic cells; mDC1, myeloid DC type 1; mDC2, myeloid DC type 2; Mono, monocytes; Mac, macrophages; CD8, CD8+ T cells; CD4, CD4+ T cells; Treg, regulatory T cells; NK, natural killer cells; B, B cells. Marker genes: T-cell cluster, *CD3E, TRAC, and IL7R*; CD4 cluster, *CD4*; CD8 cluster, *CD8B*; Treg cluster, *FOXP3 and CTLA4*; NK cluster, *GNLY, PRF1, and XCL1*; myeloid lineage cells, *LYZ*; Mono cluster, *FCGR3A and S100A8*; Mac cluster, *CD14, PLTP, MRC1, NLRP3 and IL1B*; mDC1 cluster, *XCR1*; mDC2 cluster, *FCER1A and CD1C*; pDC cluster, *TNFRSF21*; B cluster, *CD79A*. Related to *Figure 1*.


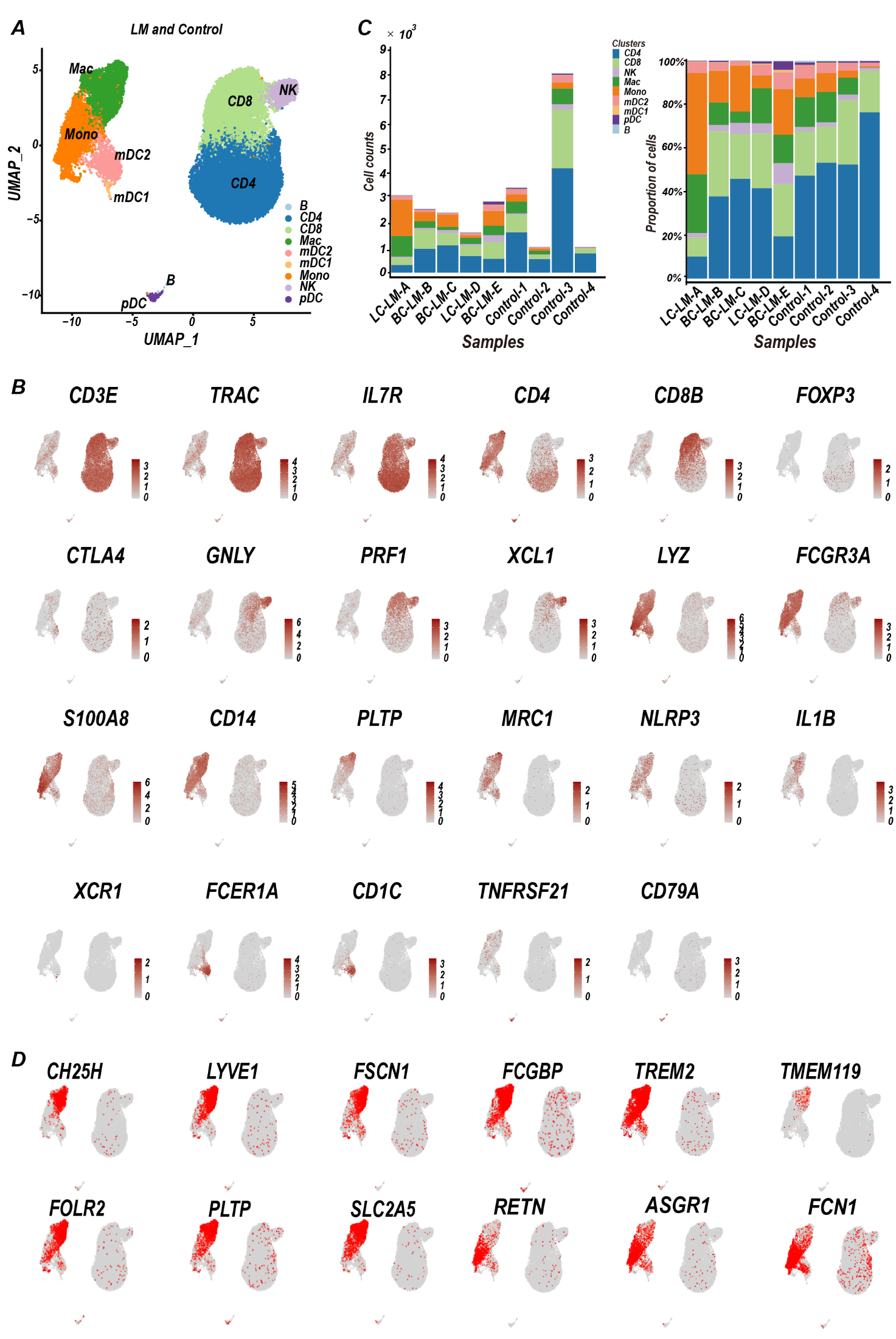


FIGURE S2 Macrophages in control samples show M1 or M2-polarized phenotype

(A) Unsupervised trajectory of monocytes and macrophages state transitions in control CSF samples. The branched trajectory was colored by cell states (state 1-state 5, S1-S5), cell subsets, and pseudo-time. (B) Relative expression map of genes specific to cell states (S1-S5). Selected genes in each state are indicated on the right. (C) Unsupervised trajectory of monocytes and macrophages at state 1 (S1) and state 4 (S4) shown on the *Figure S2A*. The branched trajectory was colored by cell states (state 6-state 10, S6-S10), cell subsets, and pseudo-time. (D) Relative expression map of genes specific to cell states (S6-S10). Selected genes in each state are indicated on the right. Marker genes: Pro-inflammatory macrophages*,* *CCL3, CCL4, CCL4L2, CCL3L3, CCL8, CXCL8;* anti-inflammatory macrophages*, APOE, APOC1, APOC2, TREM2, SPP1, MARCO, SEPP1.* Related to *Figure 2*.


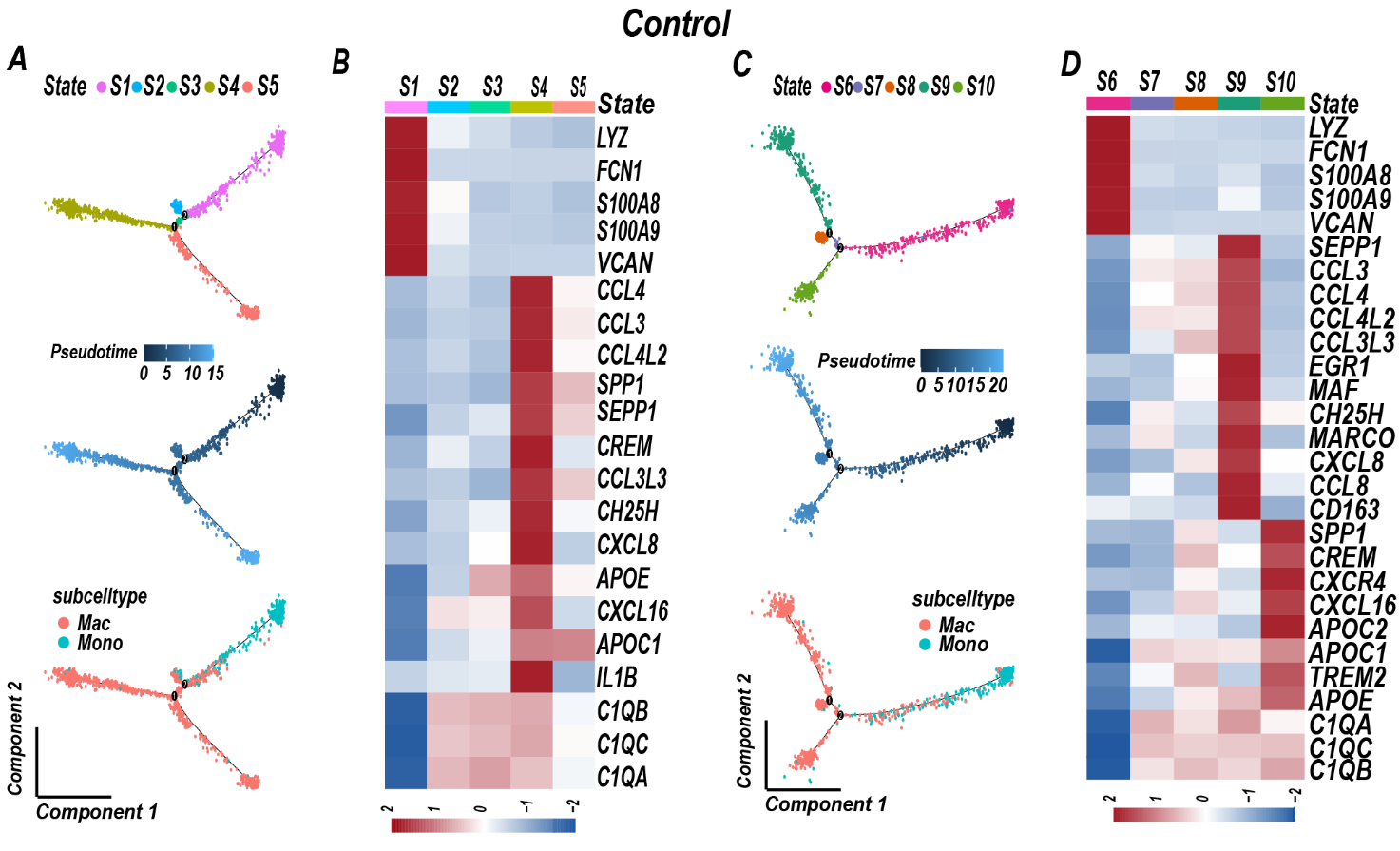


FIGURE S3 Heatmap showing the relative mean expression map of selected marker genes associated with T cell functionality in each cell cluster of leptomeningeal metastases (LM, left) and control (right) CSF samples. Marker genes: T cells (T)*, CD3D, CD3E, CD3G;* CD8+ T cells (CD8)*, CD8A, CD8B;* CD4+ T cells (CD4)*, CD4, IL7R;* Cytotoxic T cells (Cytotoxic)*, GZMA, GNLY, GZMB, GZMK, IFNG, NKG7;* Exhausted T cells (Exhausted)*, LAG3, TIGIT, PDCD1, HAVCR2;* Naïve T cells (Naïve)*, TCF7, SELL, LEF1, CCR7;* regulatory T cells (Treg)*,* *FOXP3, IL2RA, TNFRSF4, TNFRSF18.* C1-C10, cluster 1 - cluster 10, corresponding to *Figure 3A*. Related to *Figure 3*.


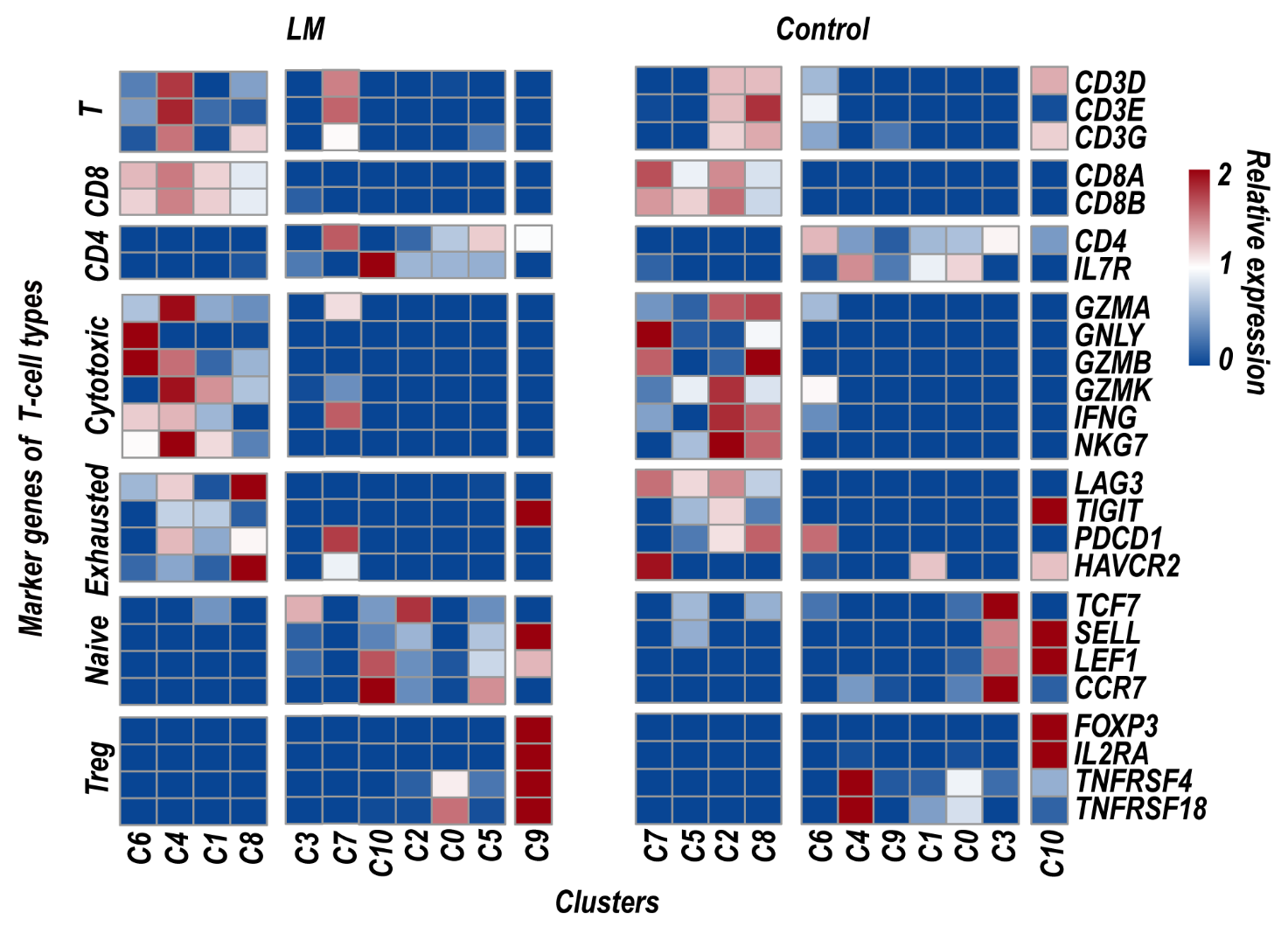


FIGURE S4 Annotation of CSF-CTCs by gene expression. (A) UMAP showing patient-derived CSF cells of three breast cancer patients with leptomeningeal metastases (BC-LM-B, BC-LM-C, BC-LM-E, GSE150681). (B) Feature plots demonstrating the expression of epithelial cell marker genes EPCAM, CDH1, KRT18 and KRT8 on the UMAP plot. (C) InferCNV plot showing diverse chromosomal copy number variation (CNVs) in the CSF-CTCs from BC-LM-B, BC-LM-C, BC-LM-E CSF samples. Normal immune cells (NK, CD4, CD8) were used as controls. Cluster key: CSF-CTCs, cerebrospinal fluid circulating tumor cells; pDC, plasmacytoid dendritic cells; mDC1, myeloid DC type 1; mDC2, myeloid DC type 2; Mono, monocytes; Mac, macrophages; CD8, CD8+ T cells; CD4, CD4+ T cells; NK, natural killer cells; B, B cells. Related to Figure 4.

***
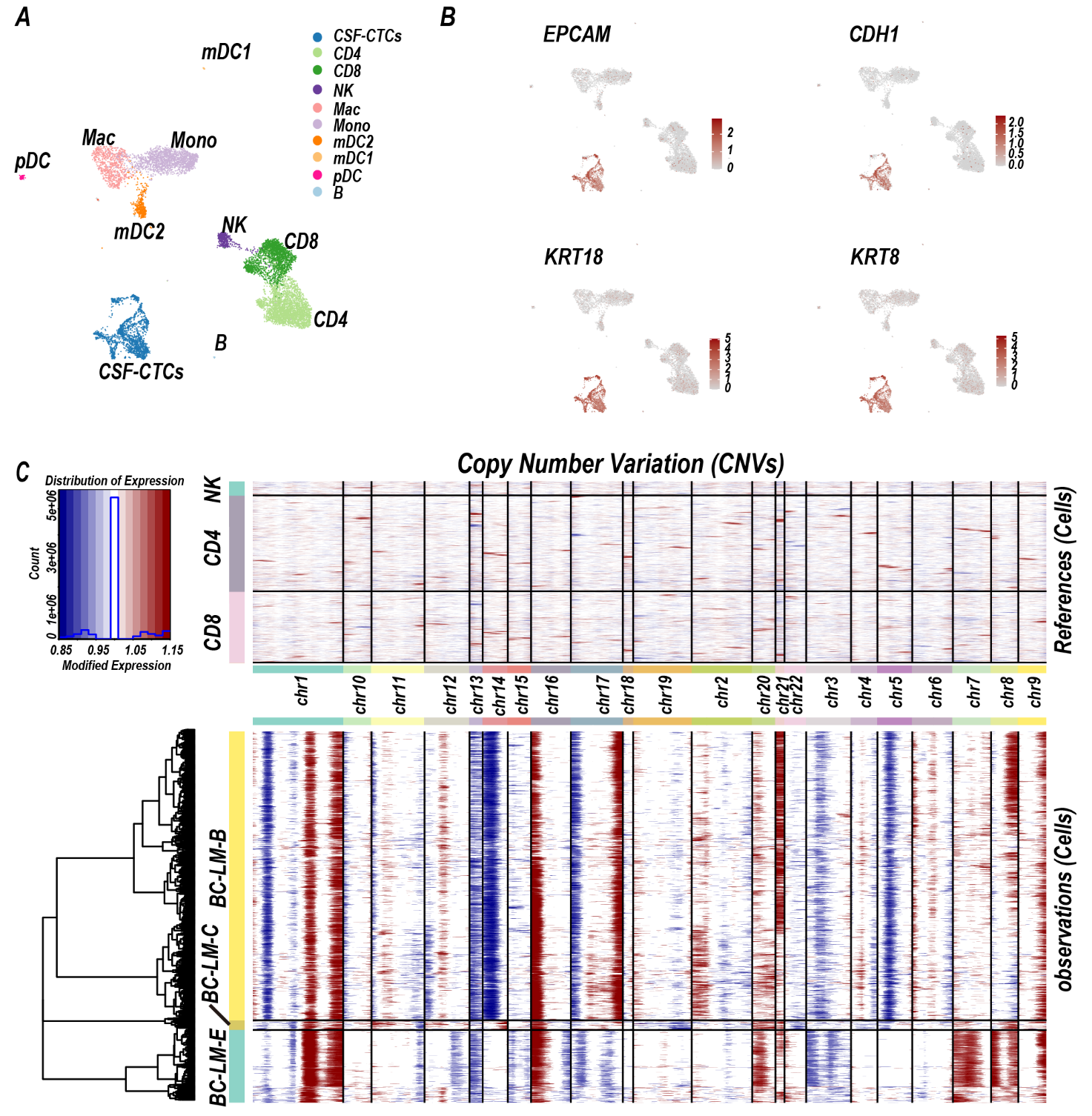
***

FIGURE S5 Heatmap of the 50 gene-expression profiles of PAM50 model in five subtype-specific CSF-CTCs. CSF sample ID: three breast cancer patients with leptomeningeal metastases (BC-LM-B, BC-LM-C, BC-LM-E, GSE150681). Subtype ID: Normal, normal-like; Basal, basal-like; Her2+, Her2 positive; LumA, Luminal A; LumB, Luminal B. Related to *Figure 4*.

***
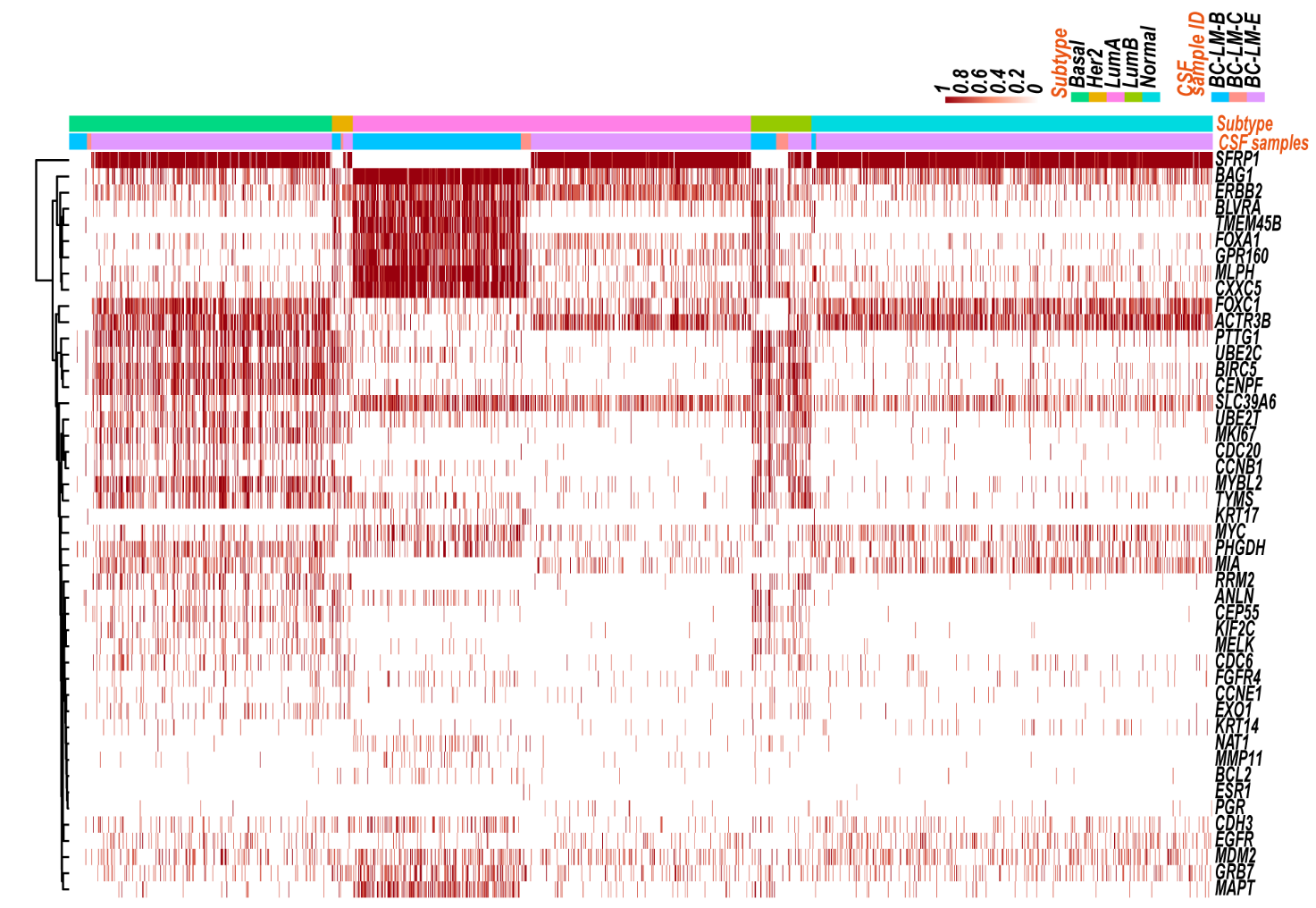
***

FIGURE S6 Cell-cell interaction network in CSF samples of five leptomeningeal metastases patients. (A) Cell-cell interaction network between different cell types. The node size represents the number of interactions. The line thickness represents the number of significant ligand-receptor interactions in two cell types. (B) Heat map depicting the number of significant interactions between cell clusters. Cluster key: pDC, plasmacytoid dendritic cells; mDC1, myeloid DC type 1; mDC2, myeloid DC type 2; Mono, monocytes; Mac, macrophages; CD8, CD8+ T cells; CD4, CD4+ T cells; NK, natural killer cells; B, B cells; CSF-CTCs, cerebrospinal fluid circulating tumor cells from five LM patients. Related to *Figure 4*.


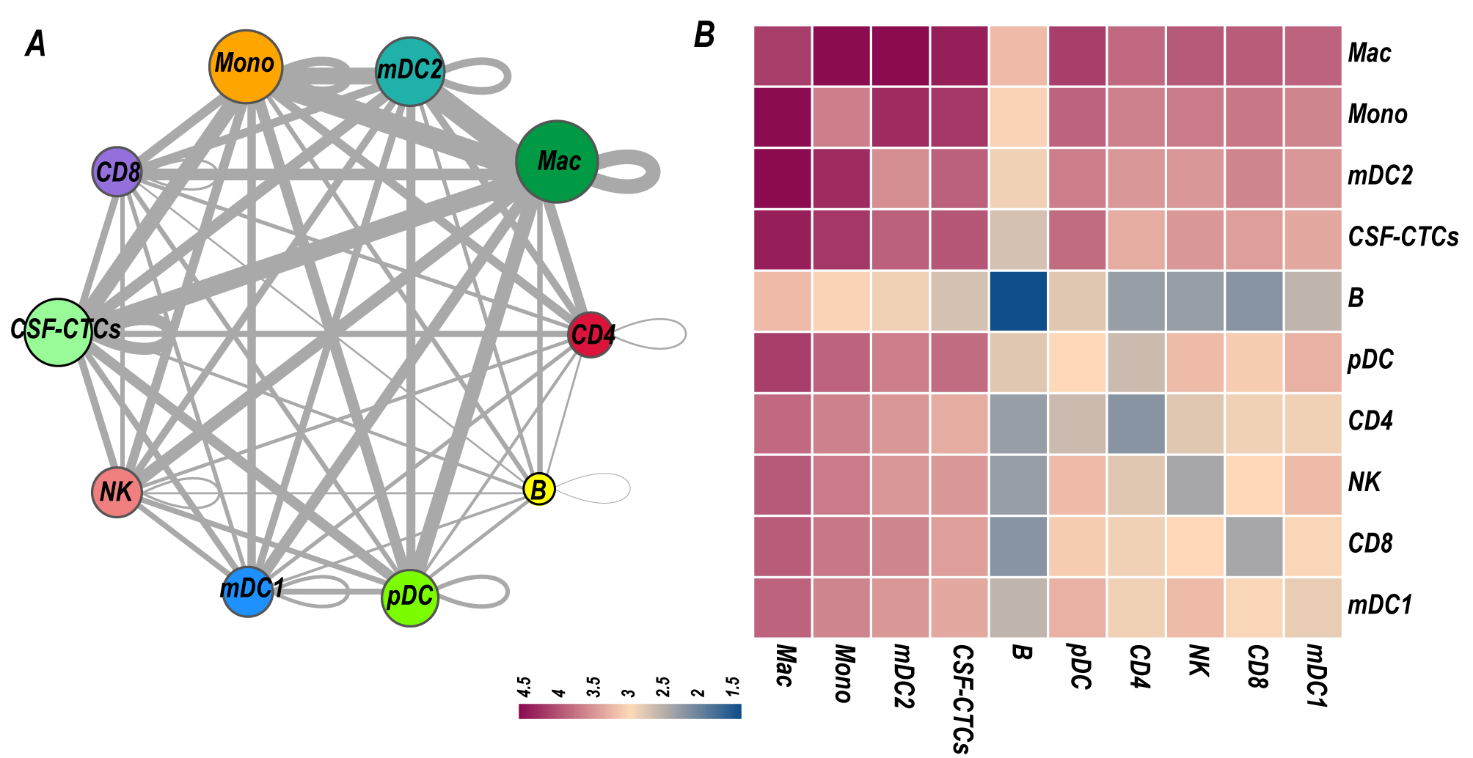


FIGURE S7 Heatmap depicting significant mean of interactions between macrophages (Mac|Basal, Mac|LumA, Mac|LumB, Mac|Her2, Mac|Normal) or monocytes (Mon|Basal, Mon|LumA, Mon|LumB, Mon|Her2, Mon|Normal) and five subtype-specific CSF-CTCs. Subtype key: Normal, normal-like; Basal, basal-like; Her2+, Her2 positive; LumA, Luminal A; LumB, Luminal B. Mac, macrophages; Mon, monocytes. N/A, Not applicable. Related to *Figure 4*.


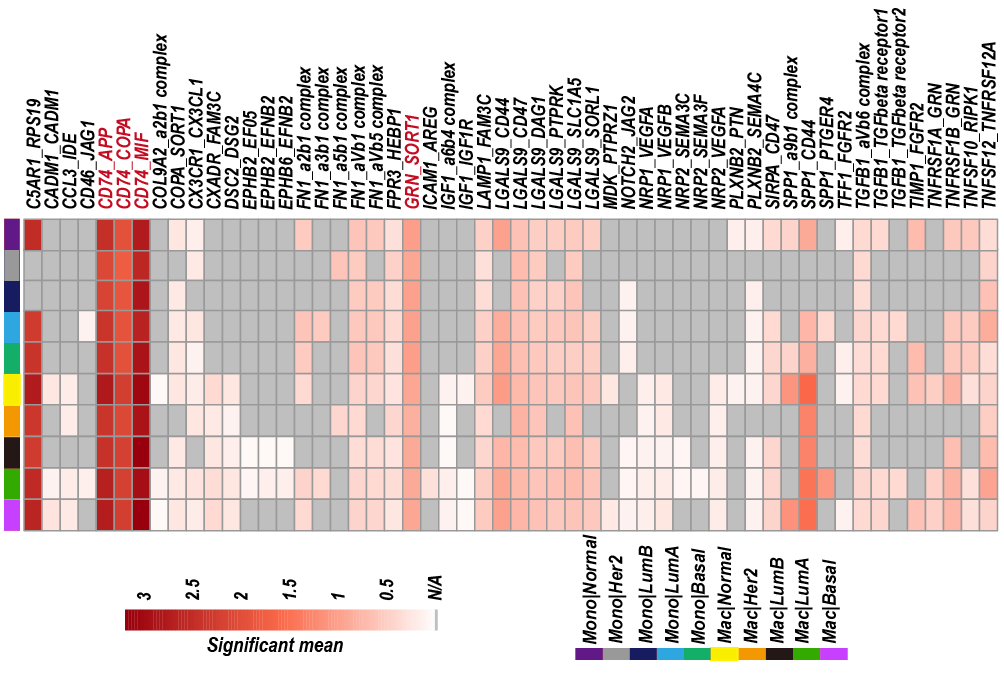


FIGURE S8 Annotation of cell clusters in BC-LM-F. (A) UMAP showing the expression of cluster marker genes (Figure 7A). Marker genes: T-cell cluster, *CD3E, TRAC, and IL7R*; CD4 cluster, *CD4*; CD8 cluster, *CD8B*; Treg cluster, *FOXP3 and CTLA4*; NK cluster, *GNLY, PRF1, and XCL1*; myeloid lineage cells, *LYZ*; Mono cluster, *FCGR3A and S100A8*; Mac cluster, *CD14, PLTP, MRC1, and IL1B*; mDC2 cluster, *FCER1A and CD1C*; B cluster, *CD79A*; epithelial cell cluster, *EPCAM, CDH1, KRT18 and KRT8*. (B) InferCNV plot showing diverse chromosomal copy number variation (CNVs) in the CSF-CTCs from BC-LM-F. Normal immune cells were used as controls. Related to Figure 7.


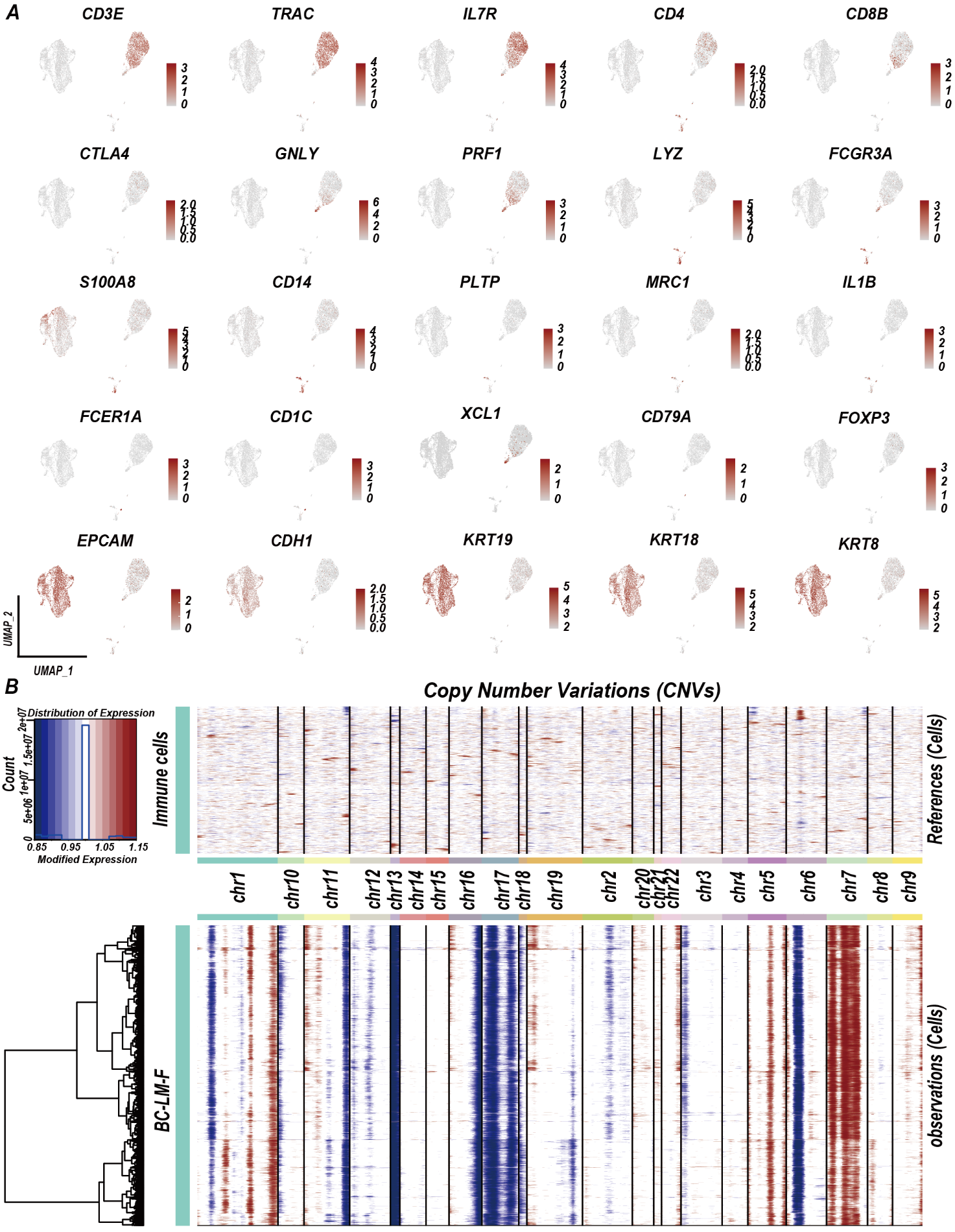


FIGURE S9 UMAP plot of CSF (cerebrospinal fluid) immune cell composition from BC-LM-F patient. Feature plots demonstrating the expression of signature genes of monocyte or macrophage cluster on the UMAP plot. Scaled expression levels are depicted using a red gradient (grey denotes lack of expression). Macrophages signature genes *CH25H, LYVE1, FSCN1, FOLR2, PLTP, SLC2A5, and FCGBP*; monocytes signature genes *RETN, ASGR1 and FCN1*. Related to Figure 7.

***
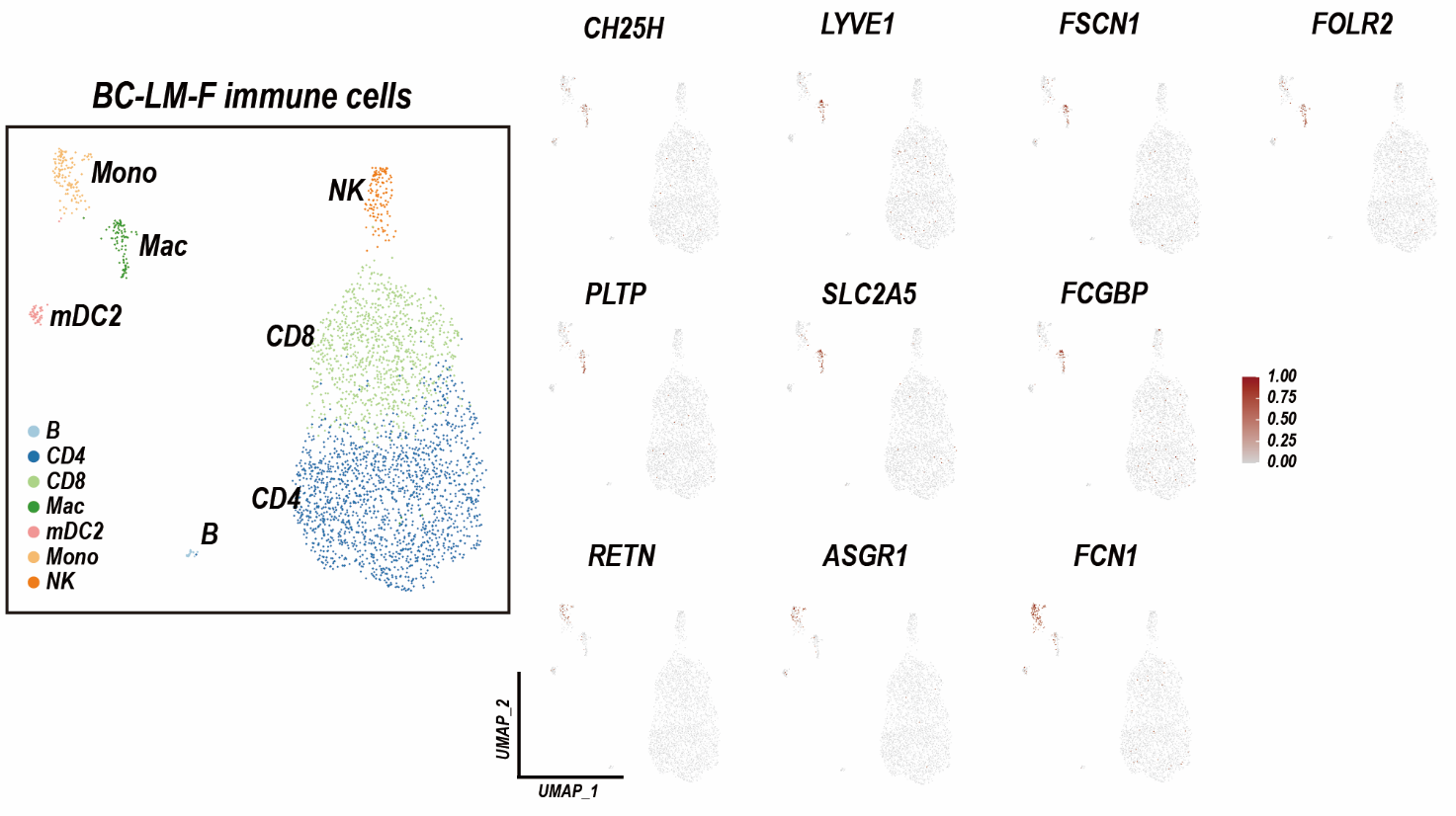
***

FIGURE S10 Macrophages and T cells in CSF derived from BC-LM-F patient. (A) Unsupervised trajectory of monocytes and macrophages state transitions in LM CSF samples. The branched trajectory was colored by cell subsets and pseudo-time. (B) Relative expression map of upregulated genes specific to monocytes or macrophages. Selected genes are indicated on the right. (C) UMAP plot of T cells, color-coded by CD4, CD8, and Treg clusters. Related to Figure 7.

***
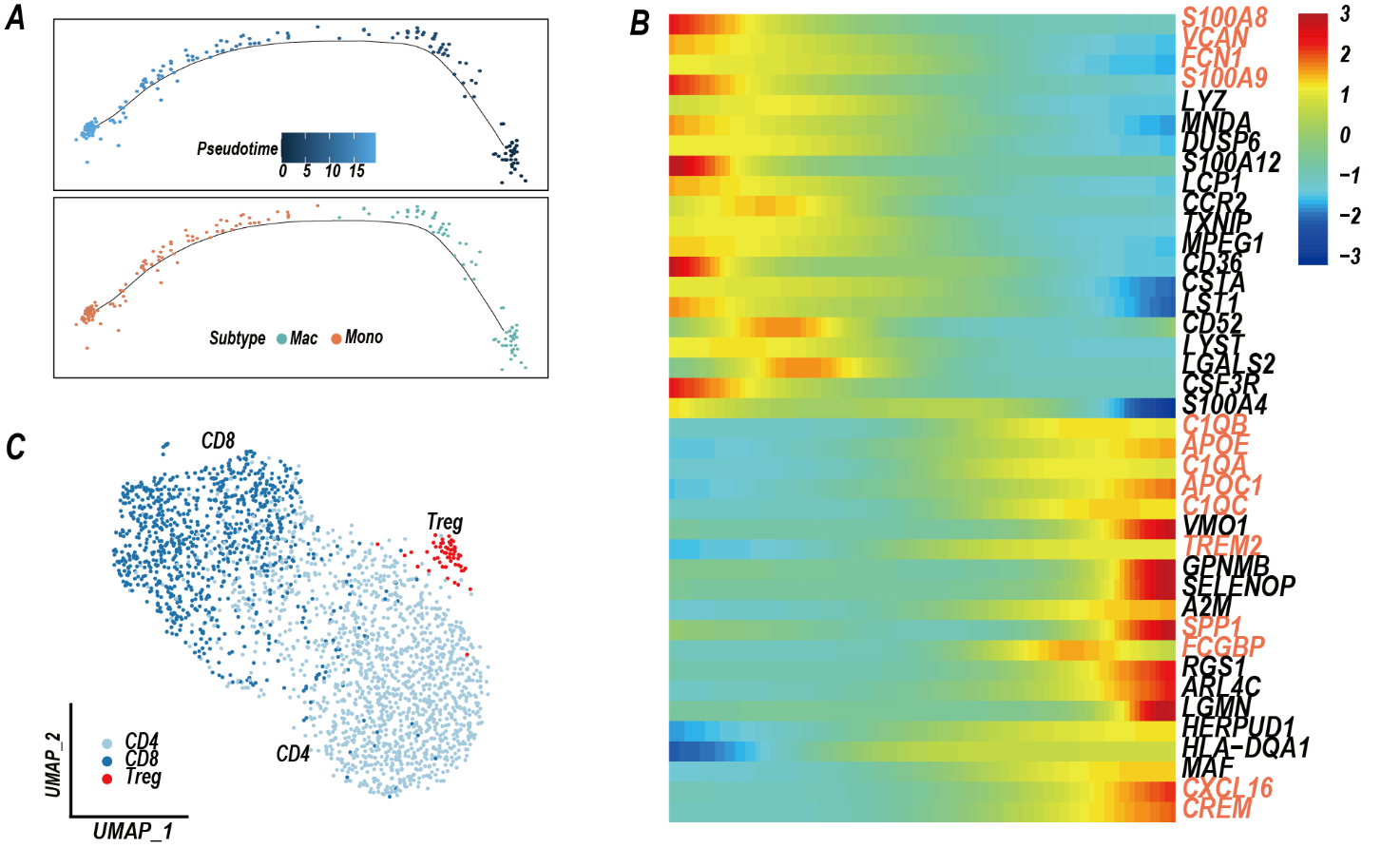
***

FIGURE S11 Heatmap depicting significant mean of interactions between five subtype-specific CSF-CTCs and macrophages (Basal|Mac, LumA|Mac, LumB|Mac, Her2|Mac, Normal|Mac) or monocytes (Basal|Mon, LumA|Mon, LumB|Mon, Her2|Mon, Normal|Mon), and between macrophages (Mac|Basal, Mac|LumA, Mac|LumB, Mac|Her2, Mac|Normal) or monocytes (Mon|Basal, Mon|LumA, Mon|LumB, Mon|Her2, Mon|Normal) and five subtype-specific CSF-CTCs, Subtype key: Normal, normal-like; Basal, basal-like; Her2+, Her2 positive; LumA, Luminal A; LumB, Luminal B. Mac, macrophages; Mon, monocytes. N/A, Not applicable. Related to Figure 7.

***
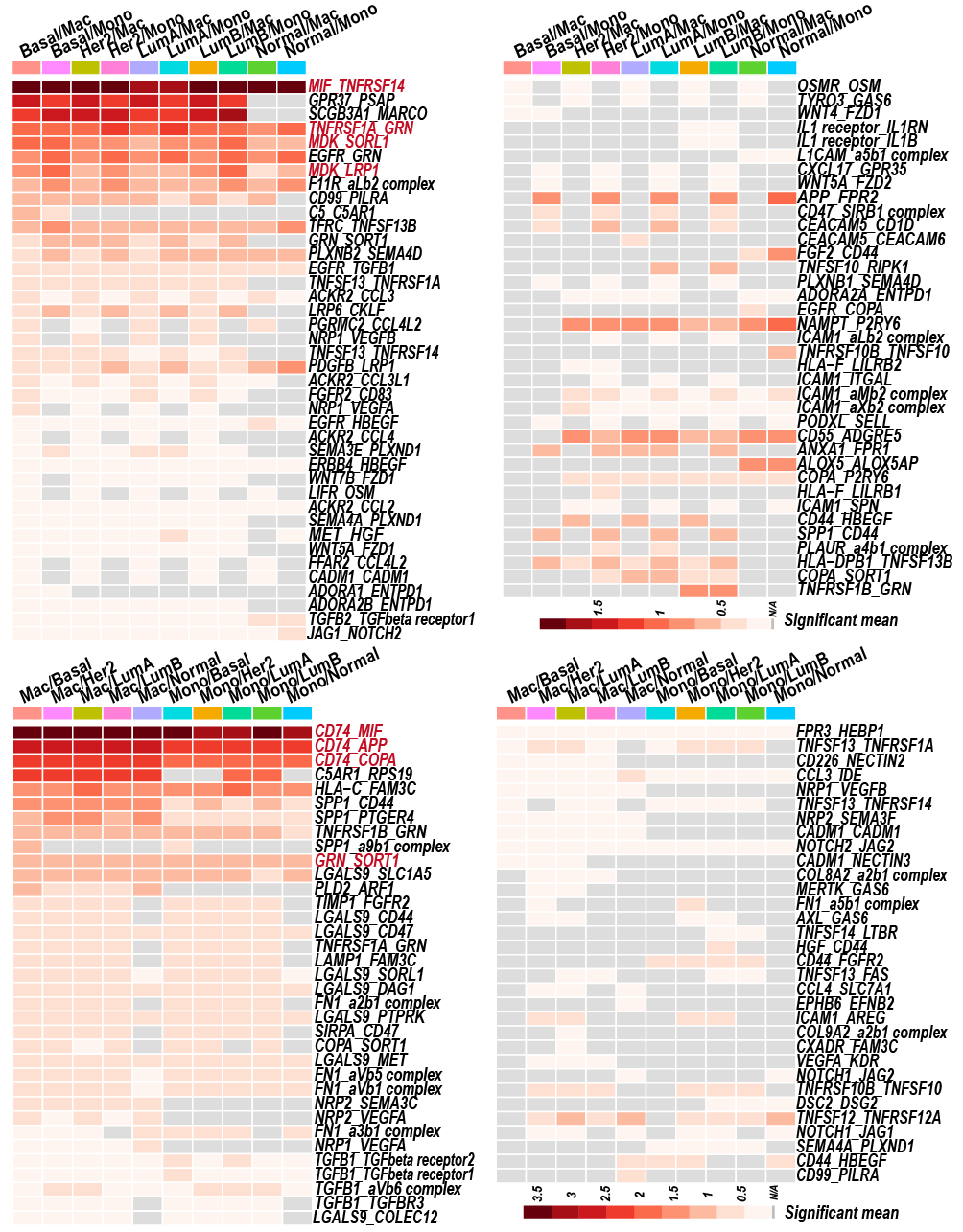
***

FIGURE S12 Characteristics of five subtype-specific CSF-CTCs from BC-LM-F sample. (A) Differences of the enrichment of the hallmark gene sets across the five subtype-specific CSF-CTCs. The colors are encoded by the mean values of the GSVA enrichment scores in the five molecular subtypes. (one-way ANOVA, ***P-value < 0.001; **P-value < 0.01; *P-value < 0.05; NS, P-value >= 0.05). (B) Venn diagram of co-expressed TF-target gene pairs of the four subtype-specific GRNs. Related to Figure 7.

***
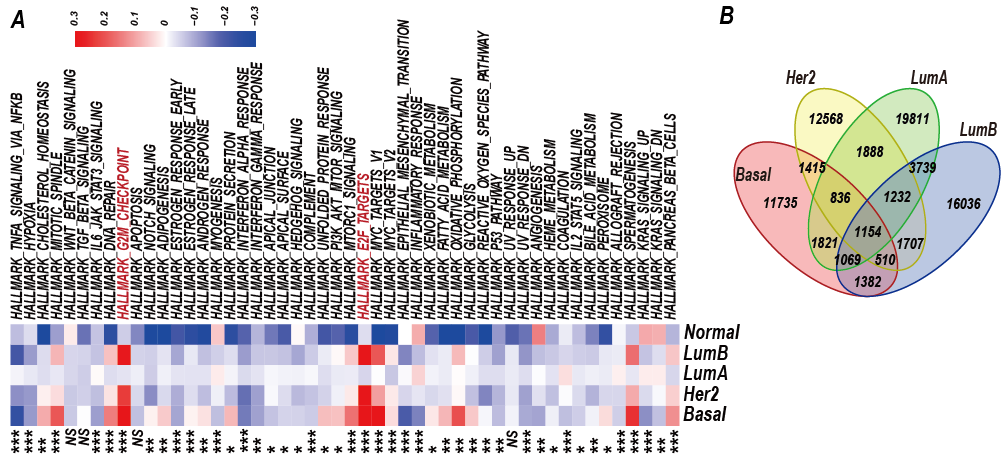
***

FIGURE S13 The difference between blood-CTCs and CSF-CTCs. (A-B) The epithelial-to-mesenchymal transition (EMT) scores of breast cancer blood-CTCs (A) and CSF-CTCs (B). (C) Venn diagram of 8 genes significantly upregulated in breast cancer CSF-CTCs compared to blood-CTCs. (D) Heat map showing the relative expression of 8 genes in breast cancer CSF-CTCs compared to blood-CTCs. CTCs ID: blood-CTC-10×, breast cancer blood CTCs sequenced by 10× genomics scRNA-seq method; blood-CTC-smart, breast cancer blood CTCs sequenced by smart-seq2 method; blood-CTC-Hydro, breast cancer blood CTCs sequenced by Hydro-seq method; BC-LM-B-CTC, BC-LM-C-CTC, BC-LM-E-CTC, BC-LM-F-CTC, CSF-CTCs from four breast cancer patients with leptomeningeal metastases.


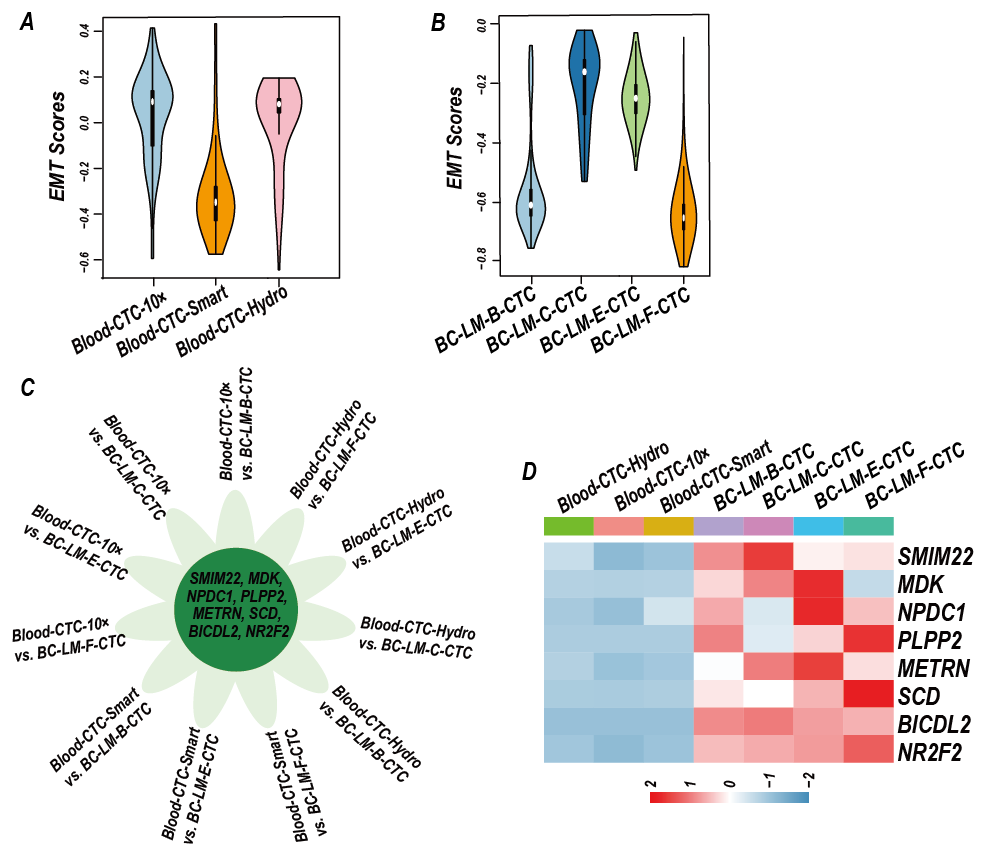


FIGURE S14 MDK expression in breast cancer.

(A) Violin plot of MDK expression in tumor tissues of breast cancer brain metastases (BC-BM) and other metastases (BC-OM), GSE14020. *P*-value (P) was calculated by two-sided Student's t test, *P* < 0.0001. (B) Expression of MDK in tumor tissues of five subtypes breast cancer, GSE19536. (C) Violin plots of MDK expression in normal breast epithelial cells of one basal cluster (N4-B) and two luminal clusters (N4-L1 and N4-L2) from one normal sample (N4, GSE113196), and five subtype-specific CSF-CTCs at single cell level. (D) Expression of MDK in BC tumor tissues (T) and adjacent non-tumorous (N) breast tissues from TCGA database. Number (num) =90, two-sided Student's t test, *P*-value < 0.0001. (E) Expression of MDK in normal tissues (N, num = 291) and BC tumor tissues (T, num = 1085) from GAPID database. (F) Violin plots of MDK expression in different cell clusters of CSF samples from four controls and six LM patients. Cluster key: pDC, plasmacytoid dendritic cells; mDC1, myeloid DC type 1; mDC2, myeloid DC type 2; Mono, monocytes; Mac, macrophages; CD8, CD8+ T cells; CD4, CD4+ T cells; NK, natural killer cells; B, B cells. Five subtype-specific CSF-CTCs: Normal, normal-like; Basal, basal-like; Her2+, Her2 positive; LumA, Luminal A; LumB, Luminal B. LC-LM-A-CTCs and LC-LM-D-CTCs, CSF-CTCs from lung cancer LM patients A and D.


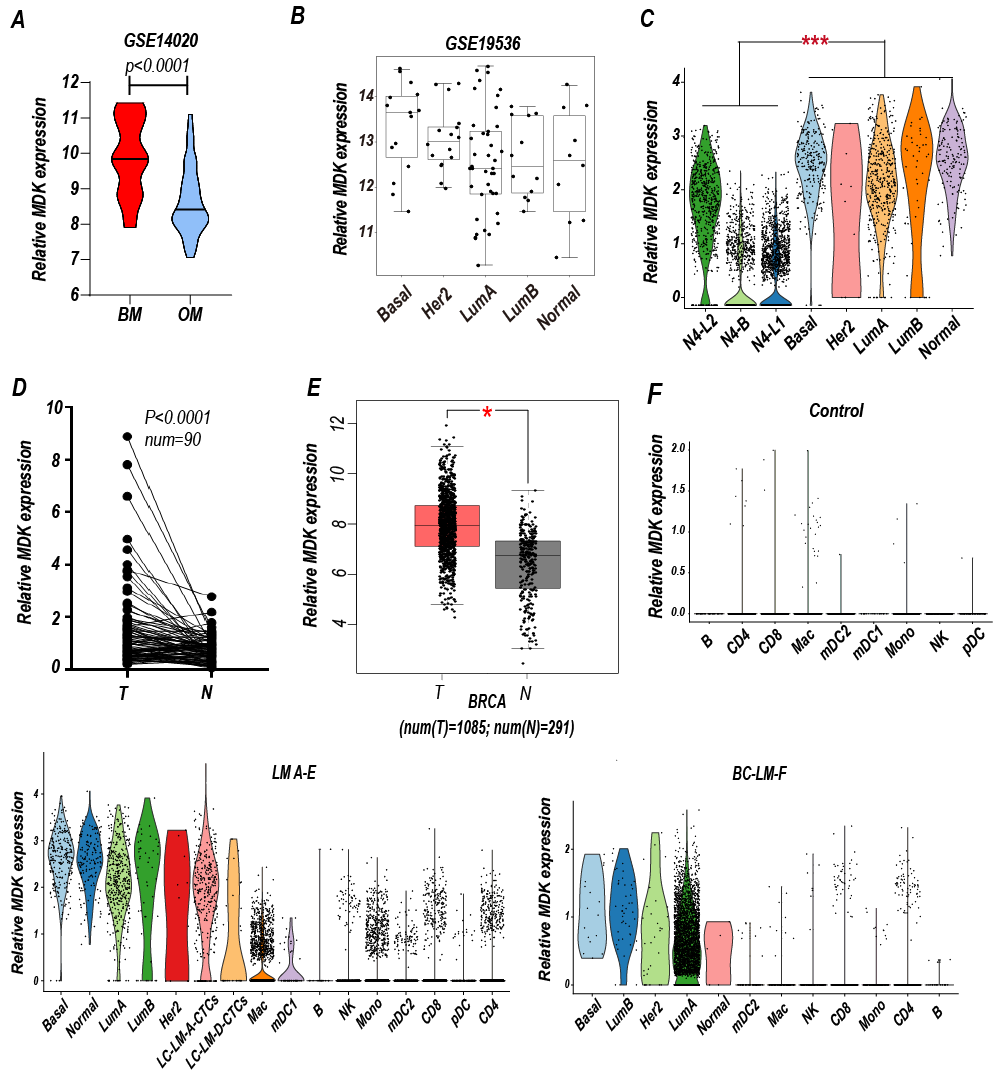


|  | | | **TABLE S1 Immune cells distribution in CSF samples of six LM patients and four controls** | | | | | | | | | |  |
| --- | --- | --- | --- | --- | --- | --- | --- | --- | --- | --- | --- | --- | --- |
|  | | **LC-LM-A** | | **BC-LM-B** | **BC-LM-C** | **LC-LM-D** | **BC-LM-E** | **BC-LM-F** | **Control- 1** | **Control- 2** | **Control-3** | **Control-**  **4** | |
| **CD4** | | 322 | | 971 | 1111 | 679 | 563 | 1602 | 1637 | 555 | 4234 | 794 | |
|  | | 10.284% | | 37.679% | 45.833% | 41.580% | 19.494% | 54.490% | 47.298% | 53.212% | 52.414% | 76.420% | |
| **CD8** | | 271 | | 774 | 499 | 411 | 692 | 928 | 704 | 172 | 2376 | 197 | |
|  | | 8.655% | | 30.035% | 20.586% | 25.168% | 23.961% | 31.565% | 20.341% | 16.491% | 29.413% | 18.961% | |
| **NK** | | 66 | | 74 | 123 | 74 | 275 | 128 | 68 | 22 | 216 | 15 | |
|  | | 2.108% | | 2.872% | 5.074% | 4.532% | 9.522% | 4.354% | 1.965% | 2.109% | 2.674% | 1.444% | |
| **Mac** | | 843 | | 266 | 124 | 263 | 377 | 92 | 472 | 144 | 627 | 1 | |
|  | | 26.924% | | 10.322% | 5.116% | 16.105% | 13.054% | 3.129% | 13.638% | 13.806% | 7.762% | 0.096% | |
| **Mono** | | 1453 | | 372 | 514 | 96 | 603 | 124 | 294 | 90 | 255 | 6 | |
|  | | 46.407% | | 14.435% | 21.205% | 5.879% | 20.880% | 4.218% | 8.495% | 8.629% | 3.157% | 0.577% | |
| **mDC1** | | 162 | | 100 | 47 | 81 | 224 | 0 | 214 | 47 | 279 | 17 | |
|  | | 5.174% | | 3.880% | 1.939% | 4.960% | 7.756% | 0.000% | 6.183% | 4.506% | 3.454% | 1.636% | |
| **mDC2** | | 5 | | 4 | 2 | 8 | 33 | 48 | 6 | 4 | 17 | 0 | |
|  | | 0.160% | | 0.155% | 0.083% | 0.490% | 1.143% | 1.633% | 0.173% | 0.384% | 0.210% | 0.000% | |
| **pDC** | | 7 | | 15 | 3 | 15 | 115 | 0 | 41 | 5 | 53 | 7 | |
|  | | 0.224% | | 0.582% | 0.124% | 0.919% | 3.982% | 0.000% | 1.185% | 0.479% | 0.656% | 0.674% | |
| **B** | | 2 | | 1 | 1 | 6 | 6 | 18 | 25 | 4 | 21 | 2 | |
|  | | 0.064% | | 0.039% | 0.041% | 0.367% | 0.208% | 0.612% | 0.722% | 0.384% | 0.260% | 0.192% | |
|  | | **3131** | | **2577** | **2424** | **1633** | **2888** | **2940** | **3461** | **1043** | **8078** | **1039** | |
|  | Cluster key: pDC, plasmacytoid dendritic cells; mDC1, myeloid DC type 1; mDC2, myeloid DC type 2; Mono, monocytes; Mac, macrophages; CD8, CD8^+^ T cells; CD4, CD4^+^ T cells; NK, natural killer cells; B, B cells.  Sample ID, Patient CSF samples: four LM patients of breast cancer (BC-LM-B, BC-LM-C, BC-LM-E, BC-LM-F), two LM patients of lung cancer (LC-LM-A, LC-LM-D), GSE150681 and GSE202501. Control CSF samples, Control 1-4, GSE138266. | | | | | | | | | | | | |
